# Coordination effects on the binding of late 3d single metal species to cyanographene†

**DOI:** 10.1039/d2cp04076j

**Published:** 2022-12-01

**Authors:** Róbert Průcha, Vítězslav Hrubý, Dagmar Zaoralová, Eva Otyepková, Veronika Šedajová, Jan Kolařík, Radek Zbořil, Miroslav Medved’, Michal Otyepka

**Affiliations:** a Department of Chemistry, Faculty of Natural Sciences, Matej Bel University Tajovského 40 974 01 Banská Bystrica Slovak Republic miroslav.medved@upol.cz; b Regional Centre of Advanced Technologies and Materials, Czech Advanced Technology and Research Institute, Palacky University Olomouc Křížkovského 511/8 77900 Olomouc Czech Republic michal.otyepka@upol.cz; c Department of Physical Chemistry, Faculty of Science, Palacký University Olomouc 17. listopadu 12 711 46 Olomouc Czech Republic; d IT4Innovations, VŠB—Technical University of Ostrava 17. listopadu 2172/15 708 00 Ostrava-Poruba Czech Republic; e Nanotechnology Centre, VŠB – Technical University of Ostrava 17. listopadu 2172/15 708 00 Ostrava-Poruba Czech Republic

## Abstract

Anchoring single metal atoms on suitable substrates is a convenient route towards materials with unique electronic and magnetic properties exploitable in a wide range of applications including sensors, data storage, and single atom catalysis (SAC). Among a large portfolio of available substrates, carbon-based materials derived from graphene and its derivatives have received growing concern due to their high affinity to metals combined with biocompatibility, low toxicity, and accessibility. Cyanographene (GCN) as highly functionalized graphene containing homogeneously distributed nitrile groups perpendicular to the surface offers exceptionally favourable arrangement for anchoring metal atoms enabling efficient charge exchange between the metal and the substrate. However, the binding characteristics of metal species can be significantly affected by the coordination effects. Here we employed density functional theory (DFT) calculations to analyse the role of coordination in the binding of late 3d cations (Fe^2+^, Fe^3+^, Co^2+^, Ni^2+^, Cu^2+^, Cu^+^, and Zn^2+^) to GCN in aqueous solutions. The inspection of several plausible coordination types revealed the most favourable arrangements. Among the studied species, copper cations were found to be the most tightly bonded to GCN, which was also confirmed by the X-ray photoelectron spectroscopy (XPS), atomic absorption spectroscopy (AAS), and isothermal titration calorimetry (ITC) measurements. In general, the inclusion of coordination effects significantly reduced the binding affinities predicted by implicit solvation models. Clearly, to build-up reliable models of SAC architectures in the environments enabling the formation of a coordination sphere, such effects need to be properly taken into account.

## Abstract 2

Coordination significantly affects the stability of single metals anchored to 2D materials. DFT applied to models including a coordination sphere and solvation effects predicts the binding affinities of late 3d cations consistently with experiment.

## Graphical abstract

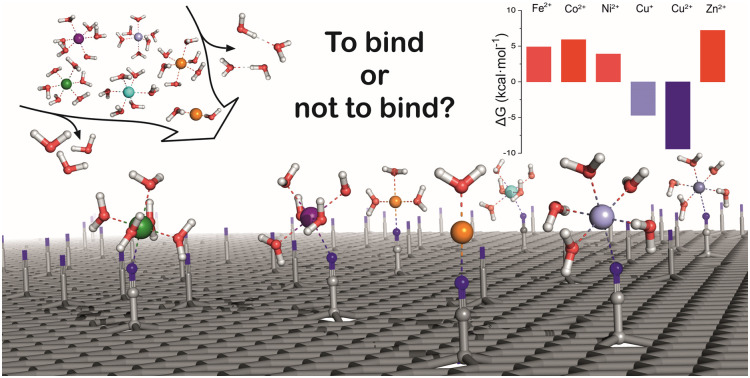

## Introduction

In the past decade, single atom catalysis (SAC) has emerged as an efficient, environmentally friendly and relatively cheap way towards the production of many commercially available chemical products.^1–4^ These days, mostly heterogenous catalysts are widely employed owing to their specificity and recyclability. The presence of rare metal elements such as Ru, Rh, Pd, Ir, and Pt is often required to achieve high catalytic activity of the material causing its high cost. Moreover, usually only the surface-exposed metal atoms are catalytically active, and thus a large fraction of the catalyst remains unused.^2^ By downsizing the metal particles to single atoms, all the active centres of the catalyst are exposed and can participate in the catalysed reaction.^3^ Nevertheless, decreasing the particle size causes a significant increase of the surface free energy^2^ which leads to the main challenge in SAC, *i.e.*, to design supporting materials that would form sufficiently strong bonds with the catalytically active single metal species (SMSs) and thus avoid undesirable migration and aggregation of SMSs into bigger particles. Careful design of the supporting material can also offer SMSs in desired oxidation and spin states.

Today, carbon-based materials are extensively studied as promising supports for SACs.^4–7^ In particular, graphene and its derivatives are attractive owing to their accessibility, low toxicity, high electric conductivity, good stability, high surface area, tunable morphologies, and high affinity to metals.^4,8–20^ In 2017, new graphene derivatives carrying –CN and –COOH groups named cyanographene (GCN) and graphene acid (GA), respectively, were synthesized for the first time employing the chemistry of fluorographene.^21^ The covalently grafted nitrile and carboxyl groups were observed to readily anchor SMSs.^9–12,16,18,19^ Kadam *et al.*^10^ reported a successful immobilization of Co(ii) cations on GCN. The GCN–Co(ii) material performed as an efficient and selective catalyst toward the oxidation of hydrazine. Bakandritsos *et al.*^9^ successfully immobilized Cu(ii) cations by nitrile groups of GCN. High-resolution X-ray photoelectron spectroscopy (HR-XPS), electron paramagnetic resonance (EPR), and X-ray absorption near edge structure (XANES) revealed that about a half of anchored Cu(ii) cations was reduced to Cu(i). The ability of GCN to reduce Cu(ii) to Cu(i) was also corroborated by density functional theory (DFT) calculations. Thanks to the presence of both Cu(i) and Cu(ii) cations, this GCN–Cu(ii)/Cu(i) mix-valence catalyst was able to activate oxygen molecules and thus excellently catalyse oxidative amine coupling reaction and selective benzylic C–H group oxidation of hydrocarbon derivatives.^9^

The metal binding to graphene derivatives significantly affects their electronic properties. Panáček *et al.*^18^ demonstrated the ability of GCN to reduce silver. Mulliken and Hirshfeld analyses indicated a significant charge transfer (*ca.* 0.5*e*) from GCN into 5s orbitals of anchored Ag^+^ cations. Langer *et al.*^11^ showed that the formation of GCN–Pt^0^ and GCN–Pt^2+^ complexes in the gas phase was associated with charge transfer to the Pt atom (Δ*q* = 0.02*e* and 1.44*e* for a neutral atom and a cation, respectively). The aqueous environment represented by an implicit solvent model caused that Pt^0^ was negligibly oxidized (Δ*q* = −0.04*e*) and lowered the reduction of Pt^2+^ (Δ*q* = 1.08*e*) after binding to GCN. Accordingly, the GCN–Pt^2+^ bond dissociation energy (BDE) was significantly lowered by the environment (from 320.9 kcal mol^−1^ in the gas phase to 91.5 kcal mol^−1^ in water) while the GCN–Pt^0^ bond strength changed only slightly (from 54.1 kcal mol^−1^ to 60.5 kcal mol^−1^).

In the extensive study by Zaoralová *et al.*^12^ on anchoring metal atoms and cations of the iron triad group (Fe, Co, Ni), light platinum group elements (Ru, Rh, Pd), and coinage metals (Cu, Ag, Au) in different oxidation states to GCN (modelled as a finite-size flake), the formation of the GCN–Me^*x*+^ bond was also usually accompanied with the metal reduction. Only in the case of zero-valent metals either negligible reduction or oxidation occurred. The degree of reduction/oxidation was explained in terms of the HOMO and LUMO energies of GCN and the respective metal atom/cation. In the case of Fe^3+^, Co^3+^, and Cu^2+^ cations coordinated on GCN, partial reduction to lower oxidation states was observed, which -in combination with their high BDEs- could be utilized for designing new mixed-valence SACs as also proposed by Bakandritsos *et al.*^9^ The GCN–Me^*x*+^ bond strength was found to be closely related to the amount of transferred charge.^12^ In the gas phase, the calculated GCN–Me^*x*+^ BDEs values reached up to 818 kcal mol^−1^ (for GCN–Au^3+^), while the aqueous environment dramatically decreased the binding affinities (with the maximum of *ca.* 289 kcal mol^−1^ also for GCN–Au^3+^). The decrease of the BDEs in water environment was attributed to solvation effects which caused the stabilization of free Me^*x*+^ species.

These studies clearly indicated the crucial role of solvation effects on the binding characteristics of GCN–Me^*x*+^. Nevertheless, due to the utilization of implicit solvent models omitting specific interactions, the conclusions drawn can only be considered qualitative, especially if the chemisorption occurs in the aqueous environment. Moreover, in the case of transition metal cations, the binding characteristics and the catalytic activity can be significantly altered by the coordination effects.^4,9,10,22–25^ It was also shown that it is crucial to distinguish the oxidation and spin states of the anchored metal species.^12,26,27^ In the models relying on periodic boundary conditions (PBC), the metal atoms are usually considered in their zero-valence state,^15,28–33^ which is valid only in certain cases, because the experimental conditions often imply the presence of coordinated cations. The treatment of such systems is more straightforward using the finite-size models,^9,10^ which avoid problems related to description of charged systems under PBC.

Here, we apply density functional theory (DFT) methods to analyse the role of coordination in the binding of late 3d cations (Fe^2+^, Fe^3+^, Co^2+^, Ni^2+^, Cu^2+^, Cu^+^, and Zn^2+^) on GCN in aqueous solutions using the finite-size approach. While the coordinating water molecules are taken into account explicitly, the surrounding aqueous environment is described by an implicit solvent model assuming the balanced description of the solute–solvent interactions for reactants and products. Theoretically predicted binding affinities of metal species to GCN are corroborated by the experimental evidence confirming the metal loading (XPS and AAS) as well as the thermal effects accompanying the anchoring of Cu cations to GCN (ITC).

## Computational details

**Fig. 1:**
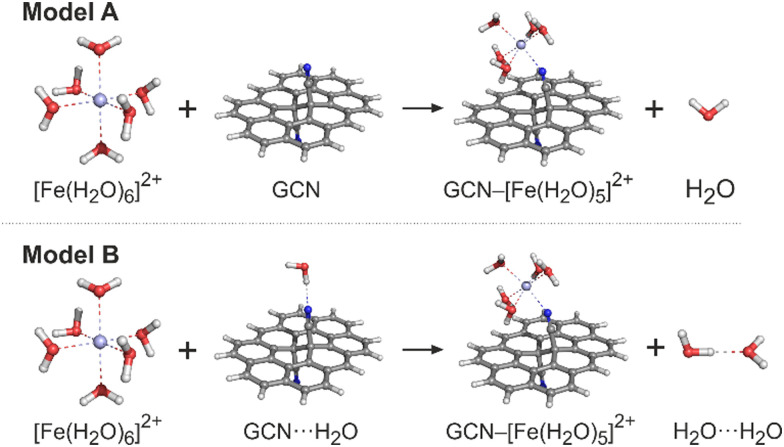
Models A and B that were applied for calculations of the GCN–Me^*x*+^ binding energies. Carbon atoms are grey, nitrogen blue, oxygen red, hydrogen white and iron light blue.

The ground state (GS) structures of all investigated species were optimized with the PBE0 method^34^ in combination with the def2-SVPP basis set. The vibrational analysis performed at the same level of theory confirmed that the optimized structures corresponded to the true minima. For the optimized structures, single-point energy (SPE) calculations were performed by applying the def2-TZVP and def2-QZVP basis sets.^35^ The choice of the exchange-correlation (XC) functional and the basis sets was based on the benchmark study by Pašteka *et al.*^36^ comparing the performance of a wide range of DFT functionals and various basis sets for evaluating the binding energies of coinage metals with lone-pair ligands taking the CCSD(T) results as the reference. The spin-unrestricted formalism was used for the open-shell system. The solvent effects were included by using the universal continuum solvation model based on solute electron density (SMD)^37^ to account for the effects of the aqueous environment. To check the sensitivity of the electronic energies to the selection of an XC functional, the ωB97X-D method was also applied in SPE calculations for comparison. Let us note that the B97-X series of XC functionals were specifically parametrized for transition-metal systems, and the ωB97X-D functional performed exceptionally well in a thorough assessment of transition metal open-shell systems.^38^ GCN was modelled as a large polycyclic aromatic hydrocarbon (PAH) molecule, ovalene (C_34_H_14_), with a single –CN functionalisation on each side. Whereas the aqua complexes of metals were fully optimized, in the case of GCN–Me species two approaches were applied. First, to mimic the semi-local rigidity of graphene sheets, all edge-atoms and sp^2^-carbons were kept frozen during constrained geometry optimizations (hereafter referred to as a frozen-sheet approach). Second, the whole GCN–Me complex was fully optimized allowing the determination of reaction Gibbs energies and the analysis of zero-point vibrational energy, enthalpic and entropic contributions (unfrozen-sheet approach). It should also be noted that some of the modelled reactions involve a change in the coordination number of a metal cation leading to an exchange (release or capture) of one (or more) water molecule(s) with the environment modelled as homogeneous medium. Also, the nitrile groups in GCN can eventually form hydrogen bonds with surrounding water molecules. To accurately address this issue is far from trivial;^39^ nevertheless, to assess the magnitude of effects related to the breaking and formation of hydrogen bonds, we employed two models (Fig. 1). In a simpler model A, the solvation of all reactive species was described using the implicit (SMD) model without considering any solute–solvent hydrogen bonds, as exemplified for an Fe^2+^ cation. In model B, an explicit water molecule was included forming a hydrogen bond with GCN and H_2_O on the reactant and product side, respectively.

Using these models, the binding energy for anchoring a metal cation to GCN corresponds to the reaction energy of the ligand exchange, *i.e.*, it is negative for a favourable formation of GCN–Me complexes. In the case of homogeneous aqua-complexes, the most stable coordination types and spin states as anticipated for weak field ligands were considered (Table S1, ESI†). For complexes involving GCN, various plausible coordination arrangements were investigated. All calculations were performed with the Gaussian16 program.^40^

## Experimental

GCN was prepared in a batch synthesis according to the method reported previously.^21^ Briefly, graphite fluoride (Sigma Aldrich, 5 g, ∼161 mmol of CF units) was dispersed in 300 mL of DMF and mixed for 2 days. The dispersion was then sonicated using Bandelin Sonorex, DT 255H type sonicator bath for 4 hours at a frequency of 35 kHz and effective power of 160 W. The dispersion was stirred for another day, after which 11.5 g (235 mmol) of NaCN (Sigma Aldrich) was added to the dispersion. The mixture was refluxed at 130 °C while stirring at 500 rpm for 3 days. After the mixture cooled down, the crude GCN was separated from the rest of the mixture by centrifugation (Sigma 16K, 13 500 rpm, 10 minutes) and washed with DMF, acetone, EtOH (all 3×), hot ultra-pure water (UPW, 1×), UPW (3×), UPW acidified with HCl (pH ∼ 2.5, 3×), and once again with UPW. This product was dispersed in UPW and dialyzed until the conductivity of the dispersion dropped below 100 μS cm^−1^. The final product was freeze-dried into dry black powder. The content of CN groups in the GCN was determined to 7.31 μmol of CN groups per 1 mg of GCN (see Fig. S1, ESI†).

For immobilization of metal species on GCN, 10 mg of GCN was dispersed in 4 mL of 30 mM aqueous solution of the relevant metal salt. For this, FeCl_2_·4H_2_O, Fe(NO_3_)_3_·9H_2_O, Co(NO_3_)_2_·6H_2_O, Ni(NO_3_)_2_·6H_2_O, and Cu(NO_3_)_2_·3H_2_O (all from Sigma Aldrich) were used. Each mixture was mixed on rotary mixer for 24 hours, after which the material was vacuum filtered on a frit using Nylon membrane filter (Whatman, pore size 0.45 μm, diameter 47 mm), re-dispersed in 4 mL of water by vigorous shaking, and filtered again. This way, each sample was washed four times. After each washing step a filtrate sample was taken for atomic absorption spectroscopy (AAS) measurement to monitor the progress of purification from the residual metal salt. After all the washing steps, each sample was dispersed in 4 mL of water. Materials prepared using Cu^2+^, Co^2+^, Ni^2+^, Fe^2+^, and Fe^3+^ salts were codenamed as GCN–Cu, GCN–Co, GCN–Ni, GCN–Fe2, and GCN–Fe3, respectively.

The metal loading of each sample was determined using AAS. Each sample was prepared by diluting 0.5 mL of the GCN–Me dispersion of known concentration to 25 mL (50×) in a 25 mL volumetric flask with aqueous 2% HNO_3_ followed by two-hour sonication. The concentration of the material in the dispersion was determined by evaporation of known volume of the material dispersion in thermal analysis instrument to precisely measure the weight of the solid residue. The dispersion was then filtered through the syringe filter (Chromafil® GF-100/15 MS, pore size 1 μm, diameter 15 mm) and the metal concentration of the filtrate was determined using AAS to estimate the metal loading on the GCN. The concentration of metals was determined by the electrothermal atomization atomic absorption spectroscopy (ETA-AAS) technique, using a graphite furnace (ContrAA 600; Analytik Jena AG, Germany). The spectrometer was equipped with a high-resolution Echelle double monochromator (spectral bandwidth, 2 pm at 200 nm). A xenon lamp was used as a continuum radiation source. For determination of metals concentration was used a standard calibration by commercial stock standards (Sigma-Aldrich).

X-ray photoelectron spectroscopy (XPS) measurements were performed by employing a PHI VersaProbe II (Physical Electronics) spectrometer using an Al Kα source (15 kV, 50 W). Samples were deposited onto the silicon holder as water-based dispersion that was let to dry prior to the measurement. The obtained spectra were evaluated and deconvoluted using the MultiPak (Ulvac – PHI, Inc.) software package. The charge correction of all the spectra was done by positioning the C 1s sp^2^ lines at 284.8 eV. Prior to the deconvolution of Cu 2p region of GCN–Cu sample, polynomial subtract of background was performed.

Isothermal titration calorimetry (ITC) measurements were carried out using Microcalorimeter TAM IV instrument (TA Instruments) employing TAM Assistant – version 2.0.175.1 (TA Instruments) measuring software. Overall, three experiments were performed, all at temperature of 25 °C. In each of them, 800 μL of GCN dispersion (twice with the concentration of 3.125 mg mL^−1^ which is equivalent to 22.84 mM in terms of CN groups, once as 7.6 mM CN solution) was titrated with Cu(NO_3_)_2_ solution by injecting 60 × 3 μL of 50 mM solution, 60 × 3 μL of 37.5 mM solution, and 50 × 4 μL of 10 mM solution, respectively. The time interval between each injection was 5 minutes. The measured data were evaluated using NanoAnalyze Software – version 3.12.0 (TA Instruments). The measured heat rate data were transformed into enthalpy-mole ratio dependencies that were fitted using the independent model.

## Results and discussion

### Structural, energetic, and charge transfer aspects of the binding

The binding of metal cations on GCN in the aqueous environment can involve significant changes in the coordination sphere of the metal including the alternations in the coordination number of ligands, their arrangement as well as the electron density redistribution between the metal and ligands giving rise to the changes in the oxidation state of the metal. All these changes determine the resulting binding affinity of a metal expressed in terms of the binding energy. Before we analyse the binding characteristics of individual metals in more details, let us comment on some general observations and methodological aspects.

**Fig. 2:**
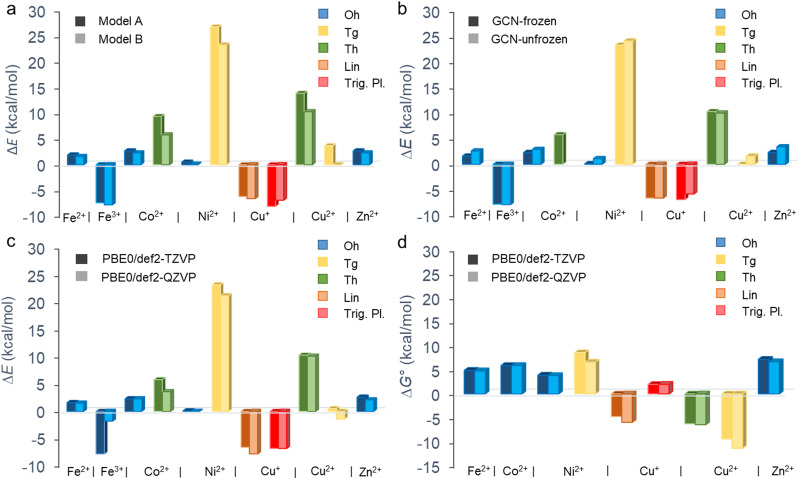
Electronic (Δ*E*) and standard Gibbs (Δ*G*°, *T* = 298.15 K) binding energies of aquated metal cations on the GCN substrate for different coordination types. Comparisons of (a) models A and B (Fig. 1) at the PBE0/def2-TZVP level using the frozen substrate, (b) results for frozen and fully relaxed GCN substrate at the PBE0/def2-TZVP level (model B), (c) Δ*E* and (d) Δ*G*° values obtained at the PBE0/def2-TZVP and PBE0/def2-QZVP levels (model B). The vibrational, thermal and entropic contributions to Δ*G*° were attained using the def2-SVPP basis set.

The binding energies largely vary depending not only on a metal but also on the coordination type (Fig. 2 and Table 1). Although the binding affinity of Fe^3+^ with the high positive charge is one of the highest, it is not the charge of the cation which rules the binding, as the two other cations with positive binding affinity (*i.e.*, negative binding energy) are Cu^+^ and Cu^2+^. Also, there is no clear preference for preserving the coordination number after the ligand exchange. Whereas the majority of cations retain the Oh coordination, GCN–Cu^2+^ favours the coordination number 4 (Tg and Th). The prediction of binding energies is to some extent sensitive to the inclusion of an extra explicit water molecule forming H-bonds out of the coordination sphere (Fig. 2(a)). Although the models A and B provide in general a qualitatively consistent picture of the binding, the latter appears to systematically predict slightly higher binding affinity (except GCN–Cu^+^ in the trigonal planar symmetry). Nevertheless, for the most stable coordination types the differences are typically below 2 kcal mol^−1^ (Table S3, ESI†). Similarly, the semi-rigidity of the substrate does not have a significant impact on the predicted binding affinity, as documented by small differences (∼1 kcal mol^−1^) between the values obtained by the frozen- and unfrozen-sheet approaches (Fig. 2(b) and Table S3, ESI†). The choice of an XC functional appears to be more critical. In general, ωB97X-D tends to increase the electronic binding affinities (except Ni^2+^ in the Tg symmetry, Table S4, ESI†), especially in the case of Oh complexes, which is probably related to better description of dispersion interactions between the substrate and tilted complex fragment (*vide infra*). However, this range-separated functional suffers from higher spin contamination compared to PBE0 (Table S4, ESI†), and therefore the results of the latter are only discussed further. The def2-TZVP basis set generally provides the binding energies in a reasonable agreement with the larger def2-QZVP basis set (Fig. 2(c)). A discrepancy encountered for GCN–Fe^3+^ is also probably related to the spin contamination issue (Table S4, ESI†). Finally, the thermodynamic feasibility of binding was evaluated based on the Δ*G*° values (Fig. 2(d)). Let us note that in the case of Fe^3+^ complexes the attained accuracy of geometry optimization did not allow performing a reliable vibrational analysis. For most cations, the inclusion of thermal and entropic contributions disfavours the anchoring to GCN, although an opposite effect is observed in the case of Cu^2+^ complexes. A deeper analysis indicates that entropy plays a major role (Table S5, ESI†); however, no particular mode type systematically dominates (Table S6, ESI†). For instance, the rotational contribution prevails in the case of Fe^2+^, Co^2+^, and Zn^2+^ complexes (Oh symmetry), whereas the translational and vibrational contributions dominate in other cases (Cu^+^ in Trig. Pl. and Cu^2+^/Ni^2+^ in Tg). From a thermodynamic point of view, the most favourable binding is predicted for Cu^+^ (Lin.) and Cu^2+^ (Th and Tg) cations; however, the low positive binding energies for Oh complexes of Fe^2+^, Ni^2+^, and Zn^2+^ cannot fully exclude some loading of these metals in real samples, taking into account uncertainties introduced by applied models and methods. Also, the negative binding affinity of Fe^3+^ (Oh) suggests the feasibility of anchoring iron(iii) species to GCN.

**Table 1:** Binding characteristics of aquated Me^*x*+^ cations anchored to GCN and ACN in water: *q*_i_ is the initial natural charge on the metal atom in the aqua-complex, *q*_f_ is the final charge on the metal atom in GCN–Me, Δ*q* = *q*_f_ − *q*_i_, *d* is the N–Me bond length (Å), and Δ*E* is the electronic binding energy (kcal mol^−1^) obtained at the PBE0/def2-QZVP level using model B

	[Me(aq)_*n*_]^*x*+^				GCN–Me		ACN–Me	
		*q*_i_	*q*_f_	Δ*q*	*d*	Δ*E*	*d*	Δ*E*
Fe^2+^	Oh	1.31	1.26	−0.05	2.24	1.5	2.20	0.6
Fe^3+^	Oh	1.80	1.32	−0.48	2.39	−1.8	—	—
Co^2+^	Oh	1.25	1.18	−0.07	2.16	2.3	2.16	−0.1
	Th	1.25	1.49	0.24	2.09	3.6	2.10	1.2
Ni^2+^	Oh	1.09	1.03	−0.06	2.07	0.0	2.06	−2.0
	Tg	1.09	1.43	0.35	2.03	21.2	—a	—a
Cu^+^	Lin	0.83	0.86	0.03	1.85	−7.8	1.85	−8.9
	Tr.	0.83	0.81	−0.02	1.86	−6.8	1.86	−7.2
Cu^2+^	Th	1.25	0.76	−0.49	1.86	10.1	—b	—b
	Tg	1.25	1.41	0.16	2.04	−1.4	2.06	−2.2
Zn^2+^	Oh	1.37	1.53	0.02	2.60	2.1	2.22	2.0

a Unstable structure relaxing towards Oh arrangement.

b Unstable structure relaxing towards Tg arrangement.

The replacement of a water molecule by a nitrile group of GCN in the metal coordination sphere usually causes only a slight increase of the electron density on the metal cation (Table 1); however, the reduction is much less pronounced than predicted by a simple implicit solvation model.^12^ Typically, the transferred charge to a metal amounts to *ca.* 0.1*e*, as found for Fe^2+^, Co^2+^, and Ni^2+^ (Oh). Somewhat enhanced reduction (*ca.* −0.5*e*) was observed in the case of Cu^2+^ (Th) bringing the total charge close to that found for Cu^+^, which was in line with a previous study on the mixed-valence nature of the GCN–Cu material.^9^ On the other hand, Cu^2+^ in Tg coordination appears to be partially oxidized (by 0.2*e*) compared to the [Cu(H_2_O)_6_]^2+^ complex. Similar increase of charge was observed for Co^2+^ (Th) and Ni^2+^ (Tg) but these coordination types are energetically unfavourable. To address the role of an extended delocalized π-conjugated system nearby the nitrile groups in GCN, the GCN–Me complexes were compared to acetonitrile (ACN) analogues. Contrary to previous findings neglecting the role of explicit coordinating water molecules,^12^ the binding affinity of metals as well as N–Me bond lengths are very similar for both substrates (Table 1 and Fig. S27, ESI†) suggesting that the presence of strongly electron donating ligands can suppress the charge transfer from the GCN substrate to the metal. It is also worth noting that despite some indications of the relationship between the binding affinity and the N–Me bond length (*e.g.*, the shortest bond was found for the most strongly bound complexes of Cu^+^), the overall correlation of these two parameters was not confirmed.

### Iron(ii)

**Fig. 3:**
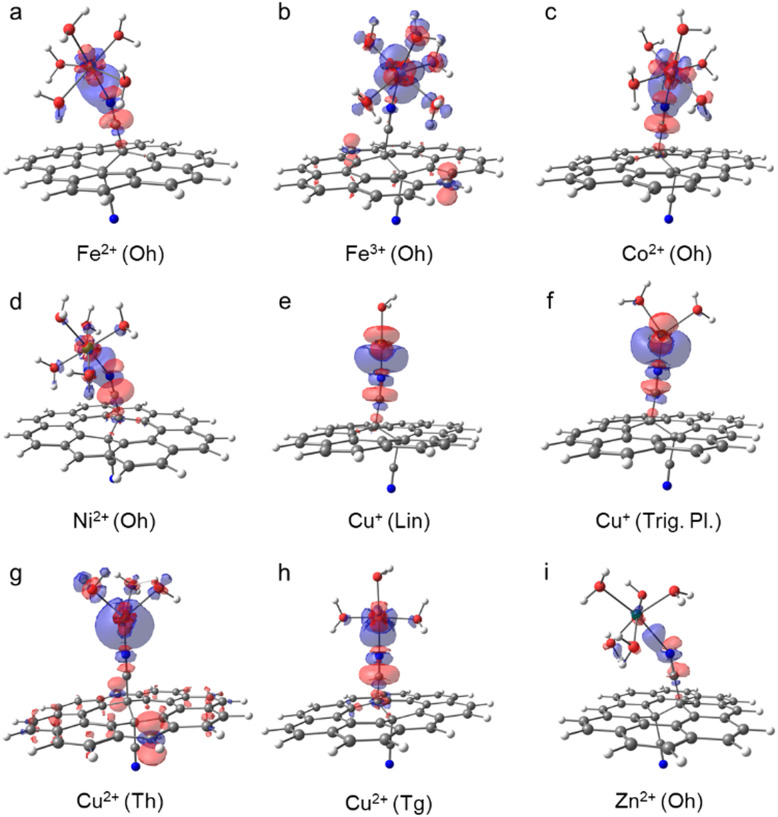
Electron density difference (EDD) plots of aquated GCN–Me complexes (blue/red regions indicate increase/decrease of the electron density induced by the binding of GCN to the aquated Me fragment; PBE0/def2-SVPP/SMD; isovalue = 0.0015).

The replacement of a water molecule in [Fe(H_2_O)_6_]^2+^ (Oh) by the GCN ligand is only slightly endergonic (Fig. 2(d)). Therefore, the loading of small amounts of Fe(ii) seems to be plausible. The ligand exchange is accompanied with a minor decrease of the atomic charge on Fe (Table 1) indicating that Fe(ii) keeps its oxidation state. The nature of the binding with GCN can be analysed in terms of the electron density difference (EDD) plots (Fig. 3(a)), where a significant increase of the electron density in the region between N and Fe atoms indicates the formation of a strong donor–acceptor σ-bond. The nitrile group acts here as an electron donor with the N lone pair interacting mainly with 4s and 4p orbitals of the central Fe(ii) atom, in which an increased population is observed (Table S8, ESI†). The NBO analysis also indicates some charge reorganization in 3d-orbitals supporting the formation of the N–Fe bond by increased population of 3d_*z*^2^_ oriented along the bond. Their mutual overlap of participating MOs is however hampered by tilting of the complex towards the GCN support owing to weak interactions with coordinating water molecules. The π-conjugated network in GCN does not appear to be directly involved in the charge transfer between the support and the metal.

### Iron(iii)

According to the electronic energy values, the anchoring of the aquated Fe^3+^ cations (Oh) to GCN support is favourable (Fig. 2(a)–(c) and Table S3, ESI†). Let us note that the Δ*G*° values for this system are not reported due to a lower accuracy of the geometry optimization of [Fe(H_2_O)_6_]^3+^ not allowing to perform the vibrational analysis. In addition, notable differences between the Δ*E* values obtained with the TZ and QZ basis sets (Fig. 2(c)) as well as the PBE0 and ωB97X-D methods were observed (Table S4, ESI†), which was probably related to slightly higher spin-contamination of the sextuplet state of GCN–[Fe(H_2_O)_5_]^3+^ for the latter (〈*S*〉^2^ = 8.80 (PBE0) *vs.* 8.83 (ωB97X-D)). The GCN–[Fe(H_2_O)_5_]^3+^ complex retains the local Oh symmetry and, similarly to GCN–[Fe(H_2_O)_5_]^2+^, it is slightly tilted towards the GCN support (Fig. 3(b)). The high binding affinity of aquated Fe^3+^ cations to GCN is predominantly attributed to charge transfer from GCN to the 3d_*xz*_ and 4p_*z*_ orbitals of Fe^3+^ (Fig. 3(b) and Table S10, ESI†) causing partial reduction of iron(iii). Interestingly, the Me–N bond is longer compared to that in GCN–[Fe(H_2_O)_5_]^2+^, despite the stronger binding of the higher oxidation state (Table 1).

### Cobalt(ii)

In water, Co^2+^ forms hexa- and tetra-coordinated aqua-complexes of Oh and Th symmetries, respectively, with the latter being less abundant,^41^ in line with our calculations predicting lower stability of Th by ∼20 kcal mol^−1^. Therefore, only the [Co(H_2_O)_6_]^2+^ (Oh) complex was considered in the following analysis. For the GCN–Co(ii) system, both Oh and Th coordination types are plausible, but again the former is notably (by ∼5 kcal mol^−1^) more stable, which is in keeping with previous XANES and extended X-ray absorption fine structure (EXAFS) measurements confirming the oxidation state +2 and the octahedral coordination of Co species in GCN–Co, respectively.^10^ The binding in the Oh symmetry is similar to that observed in the case of Fe(ii): (i) the partial atomic charge on Co(ii) remains effectively unchanged, (ii) an increase of the electron density between the N and Co atoms (Fig. 3(c)) confirms the formation of a σ-bond arising mainly from the interaction between the N lone pair and 4s and 4p orbitals of Co(ii) (Table S12, ESI†), (iii) charge reorganization in 3d orbitals of Co(ii) enhances the N–Co bonding, and (iv) the complex is tilted of towards the GCN surface. A relatively large discrepancy between the PBE0 and ωB97X-D energy values (Table S4, ESI†) arise from notable spin contamination of the quadruplet state of GCN–Co(ii) for the latter (〈*S*〉^2^ = 3.76 (PBE0) *vs.* 4.73 (ωB97X-D)).

### Nickel(ii)

For aquated GCN–Ni(ii) complexes, coordination numbers of 6 (Oh) and 4 (Tg and Th) were considered at start. However, the Th symmetry structure tends to gain an Oh arrangement, and the Tg coordination type was found much less stable than Oh (Fig. 2). Therefore, the octahedral complex as the most favourable is only analysed further in more detail. The binding energy of Ni^2+^ in this arrangement is similar to that of Fe^2+^ and Co^2+^, *i.e.*, the partial atomic charge on Ni(ii) only slightly decrease and the binding energy is close to zero, so the equilibrium between [Ni(H_2_O)_6_]^2+^ and GCN–Ni(ii) complexes can result in some loading of single Ni cations on GCN. The donor–acceptor N–Me bond is again mostly mediated by 4s, 4p, and 3d_*z*^2^_ orbitals of Ni(ii) that become more populated at the expense of the N lone pair, the nitrile group and carbon atoms in its vicinity (Fig. 3(d) and Table S16, ESI†). The Ni(ii) atom in a tilted GCN–Ni complex retains its oxidation state.

### Copper(i)

Although the coordination sphere of Cu(i) in water has not yet been fully resolved,^42,43^ our DFT calculations for different coordination numbers (2, 3, and 4) revealed that a linear [Cu(H_2_O)_2_]^+^ complex is the most probable structure, while trigonal planar and tetrahedral arrangements are unstable. On the other hand, in the case of aquated GCN–Cu(i) complexes, both coordination numbers 2 and 3 are likely, whereas the Th arrangement remains unfavourable. For both stable arrangements, the ligand exchange does not bring significant changes of the metal charge, so the copper effectively keeps its oxidation state I (Table 1). The character of bonding is also very similar in both structures: the N–Cu bond length is relatively short, in line with the high binding affinity (Fig. 2), and the increase of electron density between the Cu and N atoms (Fig. 3(e) and (f); see also increased occupancies in 4s and 4p_*z*_ orbitals on Cu in Tables S19 and S21, ESI†) arises not only from the –CN group, but also from 3d orbitals of copper.

### Copper(ii)

Upon the anchoring to GCN, the aquated Cu^2+^ complexes exhibiting Oh symmetry prefer to change the coordination number 6 to 4. Both Th and Tg structures appear to be thermodynamically stable, with a slight preference of the latter (Fig. 2), which is in line with the EPR measurements for GCN–Cu(ii) corroborating that an unpaired electron in Cu(ii) (*S* = 1/2) is localized in the d orbital (*g*_eff_ > 2.0023) and experiences a moderate tetragonal ligand field (*g*_‖_ > *g*_⊥_).^9^ While in the Th arrangement copper(ii) is reduced, in the Tg symmetry it preserves its oxidation state, which is in line with the previous study reporting the mixed-valence nature of GCN–Cu.^9^ Interestingly, the binding is significantly enhanced by the entropic contributions which overweigh relatively small enthalpy values (Table S5, ESI†). The reduction of Cu(ii) in the Th arrangement is mostly associated with the increased occupancy of the 3d_*x*^2^−*y*^2^_ orbital (Table S23, ESI†) and is supported by the electron donating capacity of GCN (Fig. 3(g)).

### Zinc(ii)

The aquated Zn^2+^ (Oh) complexes exhibit the smallest binding affinity to GCN among the studied systems with Δ*G*° being positive (∼7 kcal mol^−1^) for the most stable GCN–[Zn(H_2_O)_5_]^2+^ (Oh) arrangement. The anchoring is apparently facilitated neither by enhanced charge transfer (Δ*q* = 0.02*e*, Table 1) nor the increased occupancy of the 2p_*z*_ orbital in GCN–Me compared to [Zn(H_2_O)_6_]^2+^ (Table S26, ESI†). Also, the EDD plot for the GCN–Me complex indicates only a weak interaction between GCN and the [Zn(H_2_O)_5_]^2+^ fragment (Fig. 3(i)).

### Experimental characterization of GCN–Me samples

Table 2 summarizes the achieved metal loadings on GCN as determined by AAS in weight% and XPS in atomic%. After 4 washing steps there were still small amounts of metal ions detected in the filtrate (Fig. S2, ESI†). The successive removal of the residual Me^*x*+^ aqua complexes during the washing steps indicated the establishment of the complexation equilibria between the Me^*x*+^ aqua and GCN–Me complexes, which might enable to control the metal loading (limited by the maximum loading) by the number of washings. On the other hand, an excessive washing would lead to a complete removal of the metal. The highest loading of metal according to AAS were found for GCN–Cu (5.65 wt% of Cu) followed by GCN–Fe2 with 2.59 wt% of Fe. The loading of Fe(iii) ions (0.95 wt% of Fe) was notably smaller than that of Fe(ii), which was in contradiction with theoretically predicted binding energies (Fig. 2) indicating different chemisorption mechanism (see below). The loadings of the remaining metals were 0.84 wt% for Co in GCN–Co and 0.96 wt% for Ni in GCN–Ni. For the sake of reproducibility, GCN–Cu material was synthesized again with Cu loading determined to 5.75 wt%, in a good agreement with the previous result.

**Table 2:** Metal loadings on GCN as determined by AAS and XPS

Sample	AAS	XPS
	*c*_Me_ (wt%)	*c*_Me_ (at%)
GCN–Fe2	2.56	3.4
GCN–Fe3	0.95	1.9
GCN–Co	0.84	0.8
GCN–Ni	0.96	0.5
GCN–Cu	5.70	1.1

**Fig. 4:**
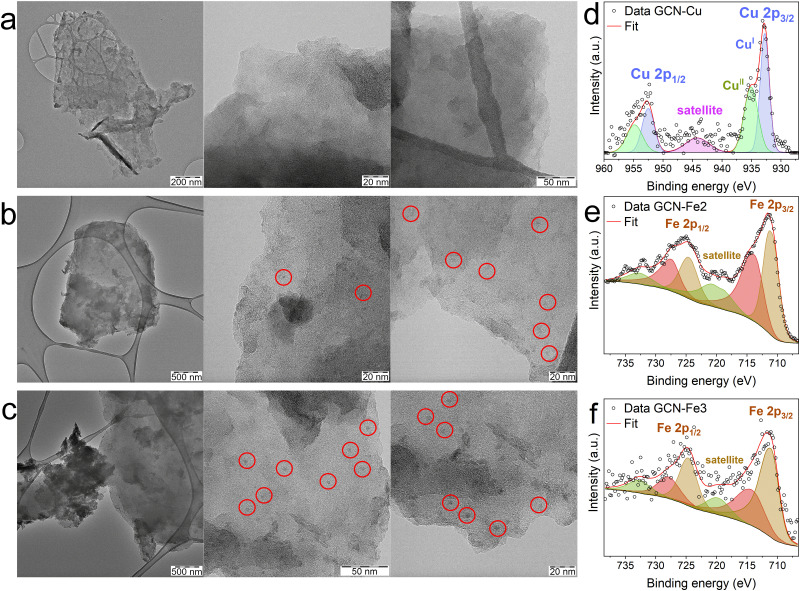
TEM images of GCN–Cu (a), GCN–Fe2 (b), and GCN–Fe3 (c). Red circles indicate the visible iron oxide nanoparticles. Deconvolution of HR-XPS spectra of Cu 2p region of GCN–Cu with polynomial subtraction of background (d), and those of Fe 2p regions of GCN–Fe2 (e) and GCN–Fe3 (f).

According to XPS analysis, the GCN–Fe samples contained higher numbers of Fe atoms, specifically 3.4 and 1.9 at% of Fe in GCN–Fe2 and GCN–Fe3 samples, respectively, compared to GCN–Cu with only 1.1 at% of Cu atoms (Table 2 and Fig. S3, ESI†). This discrepancy with respect to the AAS data was related to higher oxygen levels in the GCN–Fe samples (8.4 and 6.2 at% for GCN–Fe2 and GCN–Fe3, respectively) compared to those observed in the other GCN–Me samples (5.4–5.7 at%). This finding indicated the formation of iron oxide particles on the surface of the GCN–Fe materials, which was confirmed by TEM images (Fig. 4(b) and (c)). The absence of reflections in the XRD diffractograms of the GCN–Fe samples (Fig. S4, ESI†) indicated amorphous character of the formed nanoparticles. In comparison, GCN–Cu was probed by TEM as well showing no signs of the presence of nanoparticles (Fig. 4(a)). The difference between AAS and XPS results may thus be caused by the surface sensitivity of XPS technique. If the surface of the inspected sample is partially covered with nanoparticles, the underlying material is much less exposed to the X-ray radiation. This can result in the apparently higher detected atomic concentration of the nanoparticles’ atoms.

To further investigate the character of the metal atoms in the GCN–Fe and GCN–Cu samples, deconvoluted HR-XPS spectra of Fe 2p and Cu 2p regions were inspected. The deconvolutions of the Fe 2p region of both GCN–Fe2 and GCN–Fe3 were almost identical (Fig. 4(e) and (f)) revealing two peaks around 712 eV and 725 eV, corresponding to Fe 2p_3/2_ and Fe 2p_1/2_, respectively.^44,45^ A satellite peak located around 719 eV further indicated the Fe(iii) oxidation state.^44^ As evident from the deconvolutions, both spin–orbit components were fitted using two peaks. These peaks served more as a reference, indicating a trend, since (based on the previous studies)^46,47^ the 2p regions of transition metals should be fitted by multiple components, representing the multiplet splitting. The high similarity of the HR-XPS spectra of GCN–Fe2 and GCN–Fe3 indicated that iron atoms in both samples were present in Fe(ii) and Fe(iii) oxidation states which were not clearly distinguishable by XPS. The copper atoms in the GCN–Cu sample were found in both Cu(i) and Cu(ii) states. The mixed-valence nature of GCN–Cu was consistent with the previous findings (Fig. 4(d)).^9^

Since the Cu^2+^ ions exhibited the highest affinity towards GCN according to so-far presented theoretical and experimental observations, we probed the thermodynamics of their binding to GCN using ITC technique. The titrations of aqueous GCN dispersion with Cu(NO_3_)_2_ aqueous solutions of varying concentration provided consistent results (see Fig. S5 and Tables S28–S31, ESI†). The experimentally determined standard reaction Gibbs energy of the process (Δ*G*°) at *T* = 298.15 K equal to −6.5 ± 0.6 kcal mol^−1^ (Table 3) was found to be in a reasonable agreement with DFT calculations for the GCN–Cu^2+^ complex in its most stable Tg symmetry predicting the value −11.3 kcal mol^−1^, taking into account uncertainties of the applied computational approach estimated to ±2–3 kcal mol^−1^ (Table 3 and Table S6, ESI†). Importantly, the experiments confirmed that the binding of Cu(ii) to GCN in water is predominantly driven by the entropic contributions (Δ*S*° = 26.8 ± 0.3 kcal mol^−1^ K^−1^, −*T*Δ*S*° = −8.0 ± 0.4 kcal mol^−1^) which was in line with the theoretical predictions (Δ*S*° = 26.2 kcal mol^−1^ K^−1^, −*T*Δ*S*° = −7.8 kcal mol^−1^) for the tetragonal GCN–Cu^2+^ complex.

**Table 3:** Thermodynamic characteristics (*T* = 298.15 K) for the anchoring of Cu^2+^ ions on GCN according to experimental ITC measurements compared with theoretical values for the formation of a tetragonal (Tg) aquated GCN–Cu^2+^ complex

	Δ*G*° (kcal mol^−1^)	−*T*Δ*S*° (kcal mol^−1^)	Δ*S*° (cal mol^−1^ K^−1^)	Δ*H*° (kcal mol^−1^)
ITC	−6.5 ± 0.6	−8.0 ± 0.4	26.8 ± 1.2	1.5 ± 0.3
Tg	−11.3	−7.8	26.2	−1.6

## Conclusions

Graphene derivatives containing out-of-plane functionalities capable of acting as electron donor ligands such as GCN and GA offer unique arrangements for the efficient anchoring of single metal atoms, which leads to attractive 2D materials with high potential, *e.g.*, in single atom (electro-/photo-)catalysis. While the design of zero-valent single metal atom catalysts struggles with potential aggregation and sintering, the exploitation of metal cations in SAC faces problems of the stabilization of free cations by solvent effects and the competitive complexation of the cations with the substrate and solvent molecules or present anions. To address these issues, both solvation and coordination effects need to be properly accounted for. Here, we employed spin-polarized DFT methods to evaluate the binding affinities of a series of late 3d cations (Fe^2+^, Fe^3+^, Co^2+^, Ni^2+^, Cu^2+^, Cu^+^, and Zn^2+^) to GCN in aqueous solutions including the solvation and coordination effects. While the aqua complexes of Me^*x*+^ were assumed in their most stable arrangements and multiplicities, in the case of GCN–Me systems various coordination spheres and multiplicities were considered to determine the most favourable structures. The PBE0 and ωB97X-D methods provided mutually consistent results, although some discrepancies appeared in cases with larger spin contamination (Fe(iii), Co(ii), and Cu(ii)). Among the studied cations, Cu(i) and Cu(ii) had the highest binding affinity towards GCN in water, which was also confirmed by AAS and XPS measurements. For the GCN–Cu(ii) (Tg symmetry) being the most favourable arrangement, the calculated thermodynamic quantities were found to be in a reasonable agreement with the ITC measurements confirming that the binding is predominantly ruled by the entropy. Although Fe(iii) was also predicted to efficiently bind to GCN, the high content of oxygen revealed by XPS together with TEM images of GCN–Fe samples indicated the formation of iron oxide nanoparticles in the air exposed aqueous environment. As regards to the other metals, small positive Gibbs binding energies predicted by theory suggested the preference of cations to form free aqua complexes; nevertheless, the AAS data for sequential washings of GCN–Me samples indicated the establishment of the complexation equilibria between GCN–Me and aqua complexes enabling to reach the metal loadings suitable for specific SAC processes. Our study demonstrates that the finite-size approach considering an explicit coordination sphere of the metal species and including the environmental effects *via* the implicit solvation model is suitable for predicting the stability and favourable coordination arrangements of carbon-based materials exploiting metal cations as single atom catalytically active sites.

## Author contributions

R. P. and V. H. contributed equally. Calculations were done by R. P., D. Z., and M. M.; V. H. performed the synthesis and experimental data analysis. XPS, AAS, and ITC measurements were performed by V. Š., J. K., and E. O., respectively. The manuscript was written through contributions of all authors. The research was conceived and supervised by R. Z., M. M. and M. O. All authors have given approval to the final version of the manuscript.

## Conflicts of interest

There are no conflicts to declare.

## Supplementary Material

CP-025-D2CP04076J-s001

CP-025-D2CP04076J-s002

## References

[cit1] Cheng N., Zhang L., Doyle-Davis K., Sun X. (2019). Single-Atom Catalysts: From Design to Application. Electrochem. Energy Rev..

[cit2] Yang X.-F., Wang A., Qiao B., Li J., Liu J., Zhang T. (2013). Single-Atom Catalysts: A New Frontier in Heterogeneous Catalysis. Acc. Chem. Res..

[cit3] Liang S., Hao C., Shi Y. (2015). The Power of Single-Atom Catalysis. ChemCatChem.

[cit4] Gawande M. B., Fornasiero P., Zbořil R. (2020). Carbon-Based Single-Atom Catalysts for Advanced Applications. ACS Catal..

[cit5] Qi K., Chhowalla M., Voiry D. (2020). Single atom is not alone: Metal–support interactions in single-atom catalysis. Mater. Today.

[cit6] Wang J., Kong H., Zhang J., Hao Y., Shao Z., Ciucci F. (2021). Carbon-based electrocatalysts for sustainable energy applications. Prog. Mater. Sci..

[cit7] Zhang H., Liu G., Shi L., Ye J. (2018). Single-Atom Catalysts: Emerging Multifunctional Materials in Heterogeneous Catalysis. Adv. Energy Mater..

[cit8] Georgakilas V., Perman J. A., Tuček J., Zbořil R. (2015). Broad Family of Carbon Nanoallotropes: Classification, Chemistry, and Applications of Fullerenes, Carbon Dots, Nanotubes, Graphene, Nanodiamonds, and Combined Superstructures. Chem. Rev..

[cit9] Bakandritsos A., Kadam R. G., Kumar P., Zoppellaro G., Medved’ M., Tuček J., Montini T., Tomanec O., Andrýsková P., Drahoš B., Varma R. S., Otyepka M., Gawande M. B., Fornasiero P., Zbořil R. (2019). Mixed-Valence Single-Atom Catalyst Derived from Functionalized Graphene. Adv. Mater..

[cit10] Kadam R. G., Zhang T., Zaoralová D., Medveď M., Bakandritsos A., Tomanec O., Petr M., Zhu Chen J., Miller J. T., Otyepka M., Zbořil R., Asefa T., Gawande M. B. (2021). Single Co-Atoms as Electrocatalysts for Efficient Hydrazine Oxidation Reaction. Small.

[cit11] Langer R., Fako E., Błoński P., Vavrečka M., Bakandritsos A., Otyepka M., López N. (2020). Anchoring of single-platinum-adatoms on cyanographene: Experiment and theory. Appl. Mater. Today.

[cit12] Zaoralová D., Mach R., Lazar P., Medveď M., Otyepka M. (2021). Anchoring of Transition Metals to Graphene Derivatives as an Efficient Approach for Designing Single-Atom Catalysts. Adv. Mater. Interfaces.

[cit13] Liu J.-B., Gong H.-S., Ye G.-L., Fei H.-L. (2022). Graphene oxide-derived single-atom catalysts for electrochemical energy conversion. Rare Met..

[cit14] Majumder M., Saini H., Dědek I., Schneemann A., Chodankar N. R., Ramarao V., Santosh M. S., Nanjundan A. K., Kment Š., Dubal D., Otyepka M., Zbořil R., Jayaramulu K. (2021). Rational Design of Graphene Derivatives for Electrochemical Reduction of Nitrogen to Ammonia. ACS Nano.

[cit15] Ha M., Kim D. Y., Umer M., Gladkikh V., Myung C. W., Kim K. S. (2021). Tuning metal single atoms embedded in N_*x*_C_*y*_ moieties toward high-performance
electrocatalysis. Energy Environ. Sci..

[cit16] Blanco M., Agnoli S., Granozzi G. (2022). Graphene Acid: A Versatile 2D Platform for Catalysis. Isr. J. Chem..

[cit17] Blanco M., Mosconi D., Tubaro C., Biffis A., Badocco D., Pastore P., Otyepka M., Bakandritsos A., Liu Z., Ren W., Agnoli S., Granozzi G. (2019). Palladium nanoparticles supported on graphene acid: a stable and eco-friendly bifunctional C–C homo- and cross-coupling catalyst. Green Chem..

[cit18] Panáček D., Hochvaldová L., Bakandritsos A., Malina T., Langer M., Belza J., Martincová J., Večeřová R., Lazar P., Poláková K., Kolařík J., Válková L., Kolář M., Otyepka M., Panáček A., Zbořil R. (2021). Silver Covalently Bound to Cyanographene Overcomes Bacterial Resistance to Silver Nanoparticles and Antibiotics. Adv. Sci..

[cit19] Kolařík J., Bakandritsos A., Bad’ura Z., Lo R., Zoppellaro G., Kment Š., Naldoni A., Zhang Y., Petr M., Tomanec O., Filip J., Otyepka M., Hobza P., Zbořil R. (2021). Carboxylated Graphene for Radical-Assisted Ultra-Trace-Level Water Treatment and Noble Metal Recovery. ACS Nano.

[cit20] Zhuo H.-Y., Zhang X., Liang J.-X., Yu Q., Xiao H., Li J. (2020). Theoretical Understandings of Graphene-based Metal Single-Atom Catalysts: Stability and Catalytic Performance. Chem. Rev..

[cit21] Bakandritsos A., Pykal M., Błoński P., Jakubec P., Chronopoulos D. D., Poláková K., Georgakilas V., Čépe K., Tomanec O., Ranc V., Bourlinos A. B., Zbořil R., Otyepka M. (2017). Cyanographene and Graphene Acid: Emerging Derivatives Enabling High-Yield and Selective Functionalization of Graphene. ACS Nano.

[cit22] Kaiser S. K., Chen Z., Faust Akl D., Mitchell S., Pérez-Ramírez J. (2020). Single-Atom Catalysts across the Periodic Table. Chem. Rev..

[cit23] Li X., Rong H., Zhang J., Wang D., Li Y. (2020). Modulating the local coordination environment of single-atom catalysts for enhanced catalytic performance. Nano Res..

[cit24] Pan Y., Chen Y., Wu K., Chen Z., Liu S., Cao X., Cheong W.-C., Meng T., Luo J., Zheng L., Liu C., Wang D., Peng Q., Li J., Chen C. (2019). Regulating the coordination structure of single-atom Fe–N_*x*_C_*y*_ catalytic sites for benzene oxidation. Nat. Commun..

[cit25] Cao L., Luo Q., Liu W., Lin Y., Liu X., Cao Y., Zhang W., Wu Y., Yang J., Yao T., Wei S. (2019). Identification of single-atom active sites in carbon-based cobalt catalysts during electrocatalytic hydrogen evolution. Nat. Catal..

[cit26] Shang Y., Duan X., Wang S., Yue Q., Gao B., Xu X. (2022). Carbon-based single atom catalyst: Synthesis, characterization, DFT calculations. Chin. Chem. Lett..

[cit27] Gong Y.-N., Zhong W., Li Y., Qiu Y., Zheng L., Jiang J., Jiang H.-L. (2020). Regulating Photocatalysis by Spin-State Manipulation of Cobalt in Covalent Organic Frameworks. J. Am. Chem. Soc..

[cit28] Hossain M. D., Liu Z., Zhuang M., Yan X., Xu G., Gadre C. A., Tyagi A., Abidi I. H., Sun C., Wong H., Guda A., Hao Y., Pan X., Amine K., Luo Z. (2019). Rational Design of Graphene-Supported Single Atom Catalysts for Hydrogen Evolution Reaction. Adv. Energy Mater..

[cit29] Zhang W., Sun F.-L., Fang Q.-J., Yu Y.-F., Pan J.-K., Wang J.-G., Zhuang G.-L. (2022). Synergistic Effect of Coordination Fields and Hydrosolvents on the Single-Atom Catalytic Property in H_2_O_2_ Synthesis: A Density Functional Theory Study. J. Phys. Chem. C.

[cit30] Tian B., Ma S., Zhan Y., Jiang X., Gao T. (2021). Stability and catalytic activity to NO_x_ and NH_3_ of single-atom manganese catalyst with graphene-based substrate: A DFT study. Appl. Surf. Sci..

[cit31] Zhang J., Yang H., Liu B. (2021). Coordination Engineering of Single-Atom Catalysts for the Oxygen Reduction Reaction: A Review. Adv. Energy Mater..

[cit32] Ling C., Shi L., Ouyang Y., Zeng X. C., Wang J. (2017). Nanosheet Supported Single-Metal Atom Bifunctional Catalyst for Overall Water Splitting. Nano Lett..

[cit33] Zhao J., Chen Z. (2017). Single Mo Atom Supported on Defective Boron Nitride Monolayer as an Efficient Electrocatalyst for Nitrogen Fixation: A Computational Study. J. Am. Chem. Soc..

[cit34] Adamo C., Barone V. (1999). Toward reliable density functional methods without adjustable parameters: The PBE0 model. J. Chem. Phys..

[cit35] Weigend F., Ahlrichs R. (2005). Balanced basis sets of split valence, triple zeta valence and quadruple zeta valence quality for H to Rn: Design and assessment of accuracy. Phys. Chem. Chem. Phys..

[cit36] Pašteka L. F., Rajský T., Urban M. (2013). Toward Understanding the Bonding Character in Complexes of Coinage Metals with Lone-Pair Ligands. CCSD(T) and DFT Computations. J. Phys. Chem. A.

[cit37] Marenich A. V., Cramer C. J., Truhlar D. G. (2009). Universal Solvation Model Based on Solute Electron Density and on a Continuum Model of the Solvent Defined by the Bulk Dielectric Constant and Atomic Surface Tensions. J. Phys. Chem. B.

[cit38] Luo S., Averkiev B., Yang K. R., Xu X., Truhlar D. G. (2014). Density Functional Theory of Open–Shell Systems. The 3d-Series Transition-Metal Atoms and Their Cations. J. Chem. Theory Comput..

[cit39] Liu J., He X., Zhang J. Z. H., Qi L.-W. (2018). Hydrogen-bond structure dynamics in bulk water: insights from *ab initio* simulations with coupled cluster theory. Chem. Sci..

[cit40] Frisch M. J. , Trucks G. W., Schlegel H. B., Scuseria G. E., Robb M. A., Cheeseman J. R., Scalmani G., Barone V., Petersson G. A., Nakatsuji H., Li X., Caricato M., Marenich A. V., Bloino J., Janesko B. G., Gomperts R., Mennucci B., Hratchian H. P., Ortiz J. V., Izmaylov A. F., Sonnenberg J. L., Williams-Young D., Ding F., Lipparini F., Egidi F., Goings J., Peng B., Petrone A., Henderson T., Ranasinghe D., Zakrzewski V. G., Gao J., Rega N., Zheng G., Liang W., Hada M., Ehara M., Toyota K., Fukuda R., Hasegawa J., Ishida M., Nakajima T., Honda Y., Kitao O., Nakai H., Vreven T., Throssell K., Montgomery Jr. J. A., Peralta J. E., Ogliaro F., Bearpark M. J., Heyd J. J., Brothers E. N., Kudin K. N., Staroverov V. N., Keith T. A., Kobayashi R., Normand J., Raghavachari K., Rendell A. P., Burant J. C., Iyengar S. S., Tomasi J., Cossi M., Millam J. M., Klene M., Adamo C., Cammi R., Ochterski J. W., Martin R. L., Morokuma K., Farkas O., Foresman J. B. and Fox D. J., Gaussian 16, Revision C.01, Gaussian, Inc., Wallingford CT, 2016

[cit41] Swaddle T. W., Fabes L. (1980). Octahedral–tetrahedral equilibria in aqueous cobalt(ii) solutions at high temperatures. Can. J. Chem..

[cit42] Feller D., Glendening E. D., de Jong W. A. (1999). Structures and binding enthalpies of M^+^(H_2_O)_*n*_ clusters, M = Cu, Ag, Au. J. Chem. Phys..

[cit43] Burda J. V., Pavelka M., Šimánek M. (2004). Theoretical model of copper Cu(i)/Cu(ii) hydration. DFT and *ab initio* quantum chemical study. J. Mol. Struct..

[cit44] Fujii T., de Groot F. M. F., Sawatzky G. A., Voogt F. C., Hibma T., Okada K. (1999). *In situ* XPS analysis of various iron oxide films grown by NO_2_-assisted molecular-beam epitaxy. Phys. Rev. B: Condens. Matter Mater. Phys..

[cit45] Puthirath Balan A., Radhakrishnan S., Woellner C. F., Sinha S. K., Deng L., de los Reyes C., Rao B. M., Paulose M., Neupane R., Apte A., Kochat V., Vajtai R., Harutyunyan A. R., Chu C.-W., Costin G., Galvao D. S., Martí A. A., van Aken P. A., Varghese O. K., Tiwary C. S., Malie Madom Ramaswamy Iyer A., Ajayan P. M. (2018). Exfoliation of a non-van der Waals material from iron ore hematite. Nat. Nanotechnol..

[cit46] Gupta R. P., Sen S. K. (1975). Calculation of multiplet structure of core p-vacancy levels. II. Phys. Rev. B: Solid State.

[cit47] Biesinger M. C., Payne B. P., Grosvenor A. P., Lau L. W. M., Gerson A. R., Smart R. St. C. (2011). Resolving surface chemical states in XPS analysis of first row transition metals, oxides and hydroxides: Cr, Mn, Fe, Co and Ni. Appl. Surf. Sci..

