# Supplementary material for: Coordination effects on the binding of late 3d single metal species to cyanographene
Source: Phys Chem Chem Phys. 2022 Dec 1;25(1):286–96. doi: 10.1039/d2cp04076j (PMC9913128; doi:10.1039/d2cp04076j)
Supplement: CP-025-D2CP04076J-s002 [file CP-025-D2CP04076J-s002.pdf]

Electronic Supplementary Material (ESI) for Physical Chemistry Chemical Physics.
This journal is © the Owner Societies 2022

SUPPORTING INFORMATION

Coordination effects on the binding of late 3d single metal species to cyanographene

Róbert Průcha,†[a] Vítězslav Hrubý,†[b,c] Dagmar Zaoralová,[d] Eva Otyepková,[c] Veronika Šedajová,[b,c] Jan Kolařík,[b] Radek Zbořil,[b,e] Miroslav Medveď*[a,b] and Michal Otyepka*[b,d]

[a] *Department of Chemistry, Faculty of Natural Sciences, Matej Bel University, Tajovského 40, 974 01 Banská Bystrica, Slovak Republic.*

[b] *Regional Centre of Advanced Technologies and Materials, Czech Advanced Technology and Research Institute, Palacky University Olomouc, Křížkovského 511/8, 77900 Olomouc, Czech Republic.*

[c] *Department of Physical Chemistry, Faculty of Science, Palacký University Olomouc, 17. listopadu 12, 711 46 Olomouc, Czech Republic.*

[d] *IT4Innovations, VŠB—Technical University of Ostrava, 17. listopadu 2172/15, 708 00 Ostrava-Poruba, Czech Republic.*

[e] *Nanotechnology Centre, VŠB – Technical University of Ostrava, 17. listopadu 2172/15, 708 00 Ostrava-Poruba, Czech Republic.*

† The authors contributed equally.

* Corresponding authors: miroslav.medved@upol.cz, michal.otyepka@upol.cz

Table S1 Selected aqua-complexes of late 3*d* transition metal cations with given ground state electronic configuration, the most common multiplicity and symmetry.

**3*d* transition metal**	**Fe^{2+}**	**Fe^{3+}**	**Co^{2+}**	**Ni^{2+}**	**Cu^{+}**	**Cu^{2+}**	**Zn^{2+}**
**Multiplicity M**	5	6	4	3	1	2	1
**Configuration**	$3d^6$	$3d^5$	$3d^7$	$3d^8$	$3d^{10}$	$3d^9$	$3d^{10}$
**Symmetry of aqua-complex**	Oh	Oh	Oh	Oh	Lin	Oh	Oh

Table S2 Bonding characteristics of aqua-complexes of 3*d* transition metals bonded to GCN and acetonitrile (ACN) in water: symmetry (Symmetry) and multiplicity (M) of aqua-complex, local symmetry of the aqua-complex bonded to GCN/ACN (*Symmetry), the natural charge of Me in aqua-complex *(q_i)* and that of the metal atom in an aqua-complex bonded to GCN *(q_f)*, the difference of Mulliken charges *(Δq)*, N–Me bond length in Å *(d)*, the binding energy (in kcal/mol) computed at the PBE0/def2-TZVP level (Model A, frozen-sheet approach).

							**GCN**		**ACN**		**GCN**
Me^{x+}	***Symmetry***	***M***	****Symmetry***	***q_i***	***q_f***	**Δq**	***d***	**ΔE**	***d***	**ΔE**	**ΔE^{a}**
**Fe^{2+}**	Oh	5	Oh	1.109	1.079	–0.030	2.24	1.9	2.20	1.9	–6.1
**Fe^{3+}**	Oh	6	Oh	1.465	1.109	–0.356	2.38	-7.4	–	–	–55
**Co^{2+}**	Oh	4	Oh	1.048	1.011	–0.037	2.16	2.7	2.16	1.8	–8.7
**Co^{2+}**	Oh	4	Th	1.048	1.251	0.203	2.09	9.4	2.10	8.2	–8.7
**Ni^{2+}**	Oh	3	Oh	0.891	0.861	–0.030	2.07	0.5	2.06	–0.1	–36.6
**Ni^{2+}**	Oh	3	Tg	0.891	1.121	0.230	2.03	26.8	–[b]	–[b]	–36.6
**Cu^{+}**	Lin	1	Lin	0.598	0.651	0.053	1.85	–6.2	1.85	–7.1	–15.9
**Cu^{+}**	Lin	1	Trig. pl.	0.598	0.573	–0.025	1.86	–8.1	1.86	–7.4	–15.9
**Cu^{2+}**	Oh	2	Th	0.886	0.518	–0.368	1.86	13.9	–[c]	–[c]	–29
**Cu^{2+}**	Oh	2	Tg	0.886	1.066	0.2	2.04	3.7	2.06	4.7	–29
**Zn^{2+}**	Oh	1	Oh	1.096	1.109	0.013	2.60	2.7	2.22	2.8	–8.3

[a] Computed using the implicit solvation model (SMD) without an explicit coordination sphere at the PBE0/def2-TZVP level.[1]
[b] Unstable structure tending to reach the Oh symmetry
[c] Unstable structure tending to reach the tetragonal symmetry (Th → Tg)

Table S3 Electronic (ΔE) and Gibbs ($\Delta G°$, T = 298.15 K) binding energies (kcal/mol) for the anchoring of aquated Me complexes on GCN using equations (1) and (2) referred to as Models A and B, respectively, applying frozen- and unfrozen-sheet approaches (denoted as GCN-fr and GCN-unfr, respectively). Symmetry* refers to the local symmetry of the aqua-complex anchored to GCN. All the values were obtained at the PBE0/def2-TZVP level.

		**Model A**			**Model B**			
Me^{x+}	*Symmetry**	**ΔE GCN-fr**	**ΔE GCN-unfr**	$\Delta G°$[b]	**ΔE GCN-fr**	**ΔE GCN-unfr**	$\Delta G°$[b]	ΔE[a]
**Fe^{2+}**	Oh	1.9	3.0	6.0	1.6	2.6	4.9	–6.1
**Fe^{3+}**	Oh	–7.4[c]	–7.5[c]	–	–7.8[c]	–7.9[c]	–	–55.0
**Co^{2+}**	Oh	2.7	3.3	7.0	2.3	2.9	5.9	–8.7
**Co^{2+}**	Th	9.4	–[d]	–	5.8	–[d]	–	–8.7
**Ni^{2+}**	Oh	0.5	1.5	5.0	0.1	1.1	3.9	–36.6
**Ni^{2+}**	Tg	26.8	27.7	5.2	23.3	24.1	8.6	–36.6
**Cu^{+}**	Lin	–6.2	–6.2	–3.6	–6.6	–6.6	–4.7	–15.9
**Cu^{+}**	Trig. pl.	–8.1	–7.0	5.3	–6.9	–5.8	2.0	–15.9
**Cu^{2+}**	Th	13.9	13.6	–9.6	10.3	10.0	–6.2	–29.0
**Cu^{2+}**	Tg	3.7	5.2	–12.7	0.0	1.6	–9.4	–29.0
**Zn^{2+}**	Oh	2.7	3.8	8.3	2.3	3.4	7.2	–8.3

[a] Computed using the implicit solvation model (SMD) without an explicit coordination sphere at the PBE0/def2-TZVP level.[1]

[b] $\Delta G°$ values were obtained using a composite approach as the sum of single point electronic energy values computed at the PBE0/def2-TZVP and zero-point vibrational, thermal enthalpic, and entropic contributions obtained by frequency analysis at the PBE0/def2-SVPP level using the corresponding optimized structures.

[c] For GCN–Fe^{3+}, the electronic binding energies (ΔE) were only evaluated because only loose convergence criteria were met in the geometry optimization of the $[Fe(H_2O)_6]^{3+}$ (Oh) structure.

[d] The geometry optimization of the GCN-$[Co(H_2O)_3]^{2+}$ (Th) structure was not successful using the unfrozen-sheet approach.

Table S4 Electronic (ΔE, in kcal/mol) binding energies and S^2 values of aquated metal cations on the frozen GCN substrate for different coordination types computed using the PBE0 and ωB97X-D methods in combination with the def2-TZVP and def2-QZVP basis sets.

Cation	Symmetry	PBE0		*ω*B97X-D	
		def2-TZVP	def2-QZVP	def2-TZVP	def2-QZVP
Fe^{2+}	Oh	1.6 (6.01)	1.5 (6.01)	-3.0 (6.01)	-4.8 (6.01)
Fe^{3+}	Oh	-7.8 (8.80)	-1.8 (8.80)	-20.9 (8.84)	-19.7 (8.84)
Co^{2+}	Oh	2.3 (3.76)	2.3 (3.75)		3.3 (3.75)[a]
				-10.5 (4.73)	-10.4 (4.73)[b]
Co^{2+}	Th	5.8 (3.76)	3.6 (3.76)	0.4 (4.73)	35.4 (4.83)
Ni^{2+}	Oh	0.1 (2.00)	0.0 (2.00)	-4.7 (2.00)	-4.7 (2.00)
Ni^{2+}	Tg	23.2 (2.00)	21.2 (2.00)	47.3 (2.09)	45.0 (2.09)
Cu^{+}	Lin	-6.6 (0.00)	-7.8 (0.00)	-7.5 (0.00)	-7.7 (0.00)
Cu^{+}	Trig.Pl	-6.8 (0.00)	-6.8 (0.00)	-7.5 (0.00)	-7.4 (0.00)
Cu^{2+}	Th	10.3 (0.80)	10.1 (0.79)	6.5 (0.83)	6.4 (0.83)
Cu^{2+}	Tg	0.5 (1.37)	-1.4 (1.37)	-3.9 (1.73)	2.4 (1.83)
Zn^{2+}	Oh	2.6 (0.00)	2.1 (0.00)	-2.5 (0.00)	-2.7 (0.00)

[a] Standard SCF procedure was used. [b] Quadratically convergent SCF procedure was used.

Table S5 Thermodynamic characteristics (T = 298.15 K) for the anchoring of aquated Me complexes on GCN using the unfrozen-sheet approach in the combination with Model B employing the composite approach combining PBE0/def2-QZVP electronic energies with the ZPV, thermal enthalpic and entropic contributions obtained at the PBE0/def2-SVPP level.

	**Local symmetry of GCN-Me**	**$\Delta G°$ (kcal/mol)**	**$-T\Delta S°$ (kcal/mol)**	**$\Delta S°$ (cal/molK)**	**$\Delta H°$ (kcal/mol)**
**Fe^{2+}**	Oh	4.8	3.2	–10.8	1.7
**Co^{2+}**	Oh	5.9	3.8	–12.7	2.1
**Ni^{2+}**	Oh	3.8	3.5	–11.6	0.4
**Ni^{2+}**	Tg	6.6	–12.0	40.3	20.6
**Cu^{+}**	Lin	–5.9	2.7	–9.1	–7.4
**Cu^{+}**	Trig.Planar	2.0	7.6	–25.5	–5.6
**Cu^{2+}**	Th	–6.4	–11.9	40.0	5.7
**Cu^{2+}**	Tg	–11.3	–7.8	26.2	–1.6
**Zn^{2+}**	Oh	6.7	4.4	–14.9	2.8

Table S6 Standard reaction entropy $\Delta S°$ and its translation, rotational, and vibrational contributions for the anchoring of aquated Me complexes on GCN using the unfrozen-sheet approach in the combination with Model B obtained at the PBE0/def2-SVPP level.

	**Local symmetry of GCN-Me**	**ΔS_{transl}**	**ΔS_{rot}**	**ΔS_{vib}**	**ΔS_{tot}**
**Fe^{2+}**	Oh	–3.8	–7.3	0.3	–10.8
**Co^{2+}**	Oh	–3.8	–7.2	–1.6	–12.7
**Ni^{2+}**	Oh	–3.8	–7.0	–0.7	–11.6
**Ni^{2+}**	Tg	32.7	13.5	–5.9	40.3
**Cu^{+}**	Lin	–2.6	–1.2	–5.3	–9.1
**Cu^{+}**	Trig.Planar	–20.6	–11.4	6.7	–25.5
**Cu^{2+}**	Th	32.6	13.5	–6.1	40.0
**Cu^{2+}**	Tg	32.6	13.4	–19.8	26.2
**Zn^{2+}**	Oh	–3.9	–7.2	–3.8	–14.9

Iron (II)

Table S7 Bond distances M–L (L = O, N) of optimized structures (in Å) of $[Fe(H_2O)_6]^{2+}$, GCN–$[Fe(H_2O)_5]^{2+}$, and ACN–$[Fe(H_2O)_5]^{2+}$ complexes.

**Bond**	**System**		
	$[Fe(H_2O)_6]^{2+}$	GCN–$[Fe(H_2O)_5]^{2+}$	ACN–$[Fe(H_2O)_5]^{2+}$
Fe–O_1	2.204	2.167	2.183
Fe–O_2	2.207	2.163	2.177
Fe–O_3	2.189	2.183	2.207
Fe–O_4	2.203	2.184	2.215
Fe–O_5	2.194	2.234	2.238
Fe–O_6	2.195	–	–
Fe–N	–	**2.246**	**2.206**
C–CN	–	1.156	1.156

Table S8 Occupancies of valence orbitals of respective Fe(II) complexes (Oh), including the total occupancy as well as the natural and Mulliken charges of the central ion obtained at the PBE0/def2-SVPP level. For comparison, the total charges obtained using the def2-TZVP and def2-QZVP basis sets are reported.

Orbitals Fe^{2+}	System			Difference	
	$[Fe(H_2O)_6]^{2+}$	Fragment –$[Fe(H_2O)_5]^{2+}$	GCN–$[Fe(H_2O)_5]^{2+}$	GCN–$[Fe(H_2O)_5]^{2+}$ vs $[Fe(H_2O)_6]^{2+}$	GCN–$[Fe(H_2O)_5]^{2+}$ vs frag–$[Fe(H_2O)_5]^{2+}$
$3d_{z2}$	1.048	1.028	1.071	0.023	0.043
$3d_{x2-y2}$	1.053	1.054	1.061	0.008	0.007
$3d_{yz}$	1.630	1.008	1.011	–0.619	0.003
$3d_{xz}$	1.355	1.962	1.941	0.586	–0.021
$3d_{xy}$	1.016	1.035	1.009	0.007	–0.026
4s	0.204	0.172	0.215	0.011	0.043
$4p_x$	0.097	0.074	0.101	0.004	0.027
$4p_y$	0.093	0.079	0.107	0.014	0.028
$4p_z$	0.092	0.047	0.106	0.014	0.059
Total	6.59	6.50	6.62	**0.03**	**0.16**
Natural (DZ/ TZ/QZ)	1.40/ 1.37/1.31	1.53/ 1.53/1.49	1.37/ 1.33/1.26	**-0.03/ -0.04/-0.05**	**-0.16/ -0.20/-0.23**
Mulliken (DZ/ TZ/QZ)	1.11/ 1.19/1.40	1.24/ 1.32/1.47	1.08/ 1.09/1.39	**-0.03/ -0.09/-0.01**	**-0.16/ -0.22/-0.08**

Iron (III)

Table S9 Bond distances M–L (L = O, N) of optimized structures (in Å) of $[Fe(H_2O)_6]^{3+}$, GCN–$[Fe(H_2O)_5]^{3+}$, and ACN–$[Fe(H_2O)_5]^{3+}$ complexes.

Bond	System		
	$[Fe(H_2O)_6]^{3+}$	GCN–$[Fe(H_2O)_5]^{3+}$	ACN–$[Fe(H_2O)_5]^{3+}$
Fe–O_1	–	2.178	–
Fe–O_2	–	2.266	–
Fe–O_3	–	2.132	–
Fe–O_4	–	2.164	–
Fe–O_5	–	2.175	–
Fe–O_6	–	–	–
Fe–N	–	**2.387**	–
C–CN	–	1.155	–

Table S10 Occupancies of valence orbitals of respective Fe(III) complexes (Oh), including the total occupancy as well as the natural and Mulliken charges of the central ion obtained at the PBE0/def2-SVPP level. For comparison, the total charges obtained using the def2-TZVP and def2-QZVP basis sets are reported.

Orbitals Fe^{3+}	System			Difference	
	$[Fe(H_2O)_6]^{3+}$	Fragment –$[Fe(H_2O)_5]^{3+}$	GCN–$[Fe(H_2O)_5]^{3+}$	GCN–$[Fe(H_2O)_5]^{3+}$ vs $[Fe(H_2O)_6]^{3+}$	GCN–$[Fe(H_2O)_5]^{3+}$ vs frag–$[Fe(H_2O)_5]^{3+}$
$3d_{z2}$		1.135	1.082		–0.053
$3d_{x2-y2}$		1.194	1.064		–0.130
$3d_{yz}$		1.016	1.007		–0.009
$3d_{xz}$		1.003	1.928		0.925
$3d_{xy}$		1.033	1.011		–0.022
4s		0.206	0.206		0.000
$4p_x$		0.086	0.086		0.000
$4p_y$		0.090	0.090		0.000
$4p_z$		0.048	0.095		0.047
Total		5.81	6.57		**0.76**
Natural (DZ/ TZ/QZ)	1.91/ 1.85/1.80	2.18/ 2.20/2.17	1.41/ 1.37/1.32	**-0.50/ -0.48/-0.48**	**-0.78/ -0.83/-0.85**
Mulliken (DZ/ TZ/QZ)	1.48/ 1.46/1.95	1.78/ 1.84/2.11	1.11/ 1.15/1.42	**-0.38/ -0.31/-0.53**	**-0.67/ -0.68/-0.69**

Cobalt (Co^{2+})

A) Oh symmetry of GCN–Co^{2+}

Table S11 Bond distances M–L (L = O, N) of optimized structures (in Å) of $[Co(H_2O)_6]^{2+}$, GCN–$[Co(H_2O)_5]^{2+}$, and ACN–$[Co(H_2O)_5]^{2+}$ complexes.

Bond	System		
	$[Co(H_2O)_6]^{2+}$	GCN–$[Co(H_2O)_5]^{2+}$	ACN–$[Co(H_2O)_5]^{2+}$
Co–O_1	2.158	2.162	2.158
Co–O_2	2.140	2.156	2.159
Co–O_3	2.143	2.116	2.129
Co–O_4	2.153	2.133	2.132
Co–O_5	2.156	2.128	2.156
Co–O_6	2.159	–	–
Co–N	–	**2.165**	**2.164**
C–CN	–	1.155	1.155

Table S12 Occupancies of valence orbitals of respective Co(II) complexes (Oh), including the total occupancy as well as the natural and Mulliken charges of the central ion obtained at the PBE0/def2-SVPP level. For comparison, the total charges obtained using the def2-TZVP and def2-QZVP basis sets are reported.

Orbitals Co^{2+}	System			Difference	
	$[Co(H_2O)_6]^{2+}$	Fragment –$[Co(H_2O)_5]^{2+}$	GCN–$[Co(H_2O)_5]^{2+}$	GCN–$[Co(H_2O)_5]^{2+}$ vs $[Co(H_2O)_6]^{2+}$	GCN–$[Co(H_2O)_5]^{2+}$ vs frag–$[Co(H_2O)_5]^{2+}$
$3d_{z2}$	1.116	1.111	1.090	–0.026	–0.021
$3d_{x2-y2}$	1.124	1.111	1.141	0.017	0.030
$3d_{yz}$	1.616	1.591	1.668	0.052	0.077
$3d_{xz}$	1.647	1.672	1.759	0.112	0.087
$3d_{xy}$	1.621	1.618	1.462	–0.159	–0.156
4s	0.220	0.186	0.233	0.013	0.047
$4p_x$	0.106	0.084	0.114	0.008	0.030
$4p_y$	0.103	0.084	0.114	0.011	0.030
$4p_z$	0.103	0.052	0.123	0.020	0.071
Total	7.67	7.51	7.70	**0.05**	**0.20**
Natural (DZ/ TZ/QZ)	1.34/ 1.30/1.25	1.49/ 1.48/1.45	1.29/ 1.24/1.18	**-0.05/ -0.06/-0.07**	**-0.20/ -0.24/-0.27**
Mulliken (DZ/ TZ/QZ)	1.05/ 1.07/1.38	1.20/ 1.21/1.44	1.01/ 0.92/1.38	**-0.04/ -0.15/-0.00**	**–0.19/ -0.30/-0.06**

B) Tetrahedral symmetry of GCN–Co^{2+}

Table S13 Bond distances M–L (L = O, N) of optimized structures (in Å) of $[Co(H_2O)_4]^{2+}$, GCN–$[Co(H_2O)_3]^{2+}$, and ACN–$[Co(H_2O)_3]^{2+}$ complexes.

Bond	System		
	$[Co(H_2O)_4]^{2+}$	GCN–$[Co(H_2O)_3]^{2+}$	ACN–$[Co(H_2O)_3]^{2+}$
Co–O_1	2.060	2.043	2.053
Co–O_2	2.064	2.059	2.059
Co–O_3	2.067	2.072	2.074
Co–O_4	2.076	–	–
Co–N	–	**2.089**	**2.102**
C–CN	–	1.154	1.155

Table S14 Occupancies of valence orbitals of respective Co(II) complexes (Th), including the total occupancy as well as the natural and Mulliken charges of the central ion obtained at the PBE0/def2-SVPP level. For comparison, the total charges obtained using the def2-TZVP and def2-QZVP basis sets are reported.

Orbitals Co^{2+}	System			Difference	
	$[Co(H_2O)_6]^{2+}$	Fragment –$[Co(H_2O)_3]^{2+}$	GCN–$[Co(H_2O)_3]^{2+}$	GCN–$[Co(H_2O)_3]^{2+}$ vs $[Co(H_2O)_6]^{2+}$	GCN–$[Co(H_2O)_3]^{2+}$ vs frag–$[Co(H_2O)_3]^{2+}$
$3d_{z2}$	1.116	1.015	1.038	–0.078	0.023
$3d_{x2-y2}$	1.124	1.379	1.338	0.214	–0.041
$3d_{yz}$	1.616	1.703	1.726	0.110	0.023
$3d_{xz}$	1.647	1.678	1.700	0.053	0.022
$3d_{xy}$	1.621	1.301	1.286	–0.335	–0.015
4s	0.220	0.119	0.182	–0.038	0.063
$4p_x$	0.106	0.041	0.068	–0.038	0.027
$4p_y$	0.103	0.041	0.068	–0.035	0.027
$4p_z$	0.103	0.009	0.074	–0.029	0.065
Total	7.66	7.29	7.48	**–0.18**	**0.19**
Natural (DZ/ TZ/QZ)	1.34/ 1.30/1.25	1.71/ 1.73/1.73	1.52/ 1.50/1.49	**0.18/ 0.18/0.20**	**–0.19/ -0.23/-0.23**
Mulliken (DZ/ TZ/QZ)	1.05/ 1.07/1.38	1.44/ 1.50/1.60	1.25/ 1.15/1.38	**0.20/ 0.20/0.09**	**–0.19/ -0.34/-0.21**

Nickel (II)

A) Oh symmetry of GCN–Ni^{2+}

Table S15 Bond distances M–L (L = O, N) of optimized structures (in Å) of $[Ni(H_2O)_6]^{2+}$, GCN–$[Ni(H_2O)_5]^{2+}$, ACN–$[Ni(H_2O)_5]^{2+}$, and GCN–$[Ni(H_2O)_5]^{2+}$ (Tg) complexes.

**Bond**	**System**			
	$[Ni(H_2O)_6]^{2+}$	GCN–$[Ni(H_2O)_5]^{2+}$ (Oh symmetry)	ACN–$[Ni(H_2O)_5]^{2+}$ (Oh symmetry)	GCN–$[Ni(H_2O)_3]^{2+}$ (Tg symmetry)
Ni–O_1	2.075	2.074	2.079	2.005
Ni–O_2	2.068	2.079	2.073	2.002
Ni–O_3	2.070	2.070	2.070	2.013
Ni–O_4	2.078	2.081	2.069	–
Ni–O_5	2.074	2.070	2.069	–
Ni–O_6	2.085	–	–	–
Ni–N	–	**2.076**	**2.066**	**2.033**
C–CN	–	1.154	1.154	1.154

Table S16 Occupancies of valence orbitals of respective Ni(II) complexes (Oh), including the total occupancy as well as the natural and Mulliken charges of the central ion obtained at the PBE0/def2-SVPP level. For comparison, the total charges obtained using the def2-TZVP and def2-QZVP basis sets are reported.

Orbitals Ni^{2+}	System			Difference	
	$[Ni(H_2O)_6]^{2+}$	Fragment –$[Ni(H_2O)_5]^{2+}$	GCN–$[Ni(H_2O)_5]^{2+}$	GCN–$[Ni(H_2O)_5]^{2+}$ vs $[Ni(H_2O)_6]^{2+}$	GCN–$[Ni(H_2O)_5]^{2+}$ vs frag–$[Ni(H_2O)_5]^{2+}$
$3d_{z2}$	1.095	1.065	1.105	0.010	0.040
$3d_{x2-y2}$	1.095	1.105	1.104	0.009	–0.001
$3d_{yz}$	1.996	1.996	1.983	–0.013	–0.013
$3d_{xz}$	1.996	1.997	1.984	–0.012	–0.013
$3d_{xy}$	1.996	1.997	1.997	0.001	0
4s	0.251	0.218	0.261	0.010	0.043
$4p_x$	0.128	0.106	0.135	0.007	0.029
$4p_y$	0.130	0.101	0.140	0.010	0.039
$4p_z$	0.128	0.059	0.148	0.020	0.089
Total	8.82	8.64	8.86	**0.04**	**0.21**
Natural (DZ/ TZ/QZ)	1.18/ 1.12/1.09	1.35/ 1.33/1.31	1.14/ 1.07/1.03	**-0.04/ -0.05/-0.06**	**-0.22/ -0.26/-0.28**
Mulliken (DZ/ TZ/QZ)	0.89/ 0.79/1.23	1.05/ 0.96/1.31	0.86/ 0.65/1.22	**-0.03 -0.14/-0.01**	**–0.19/ -0.31/-0.09**

(B) Tetragonal symmetry of of GCN–Ni^{2+}

Table S17 Occupancy of valence orbitals of respective systems Ni^{2+} (Tg), including the total occupancy as well as the natural and Mulliken charges of the central ion obtained at the PBE0/def2-SVPP level. For comparison, the total charges obtained using the def2-TZVP and def2-QZVP basis sets are reported.

Orbitals Ni^{2+}	System			Difference	
	$[Ni(H_2O)_6]^{2+}$	Fragment –$[Ni(H_2O)_3]^{2+}$	GCN–$[Ni(H_2O)_3]^{2+}$	GCN–$[Ni(H_2O)_3]^{2+}$ vs $[Ni(H_2O)_6]^{2+}$	GCN–$[Ni(H_2O)_3]^{2+}$ vs frag–$[Ni(H_2O)_3]^{2+}$
$3d_{z2}$	1.095	1.146	1.192	0.097	0.046
$3d_{x2-y2}$	1.095	1.923	1.915	0.820	–0.008
$3d_{yz}$	1.996	1.997	1.988	–0.008	–0.009
$3d_{xz}$	1.996	1.993	1.983	–0.013	–0.010
$3d_{xy}$	1.996	1.024	1.023	–0.973	–0.001
4s	0.251	0.191	0.249	–0.002	0.058
$4p_x$	0.128	0.058	0.094	–0.034	0.036
$4p_y$	0.130	0.020	0.030	–0.100	0.010
$4p_z$	0.128	0.033	0.103	–0.025	0.070
Total	8.82	8.39	8.58	**–0.24**	**0.19**
Natural (DZ/ TZ/QZ)	1.17/ 1.12/1.09	1.61/ 1.63/1.64	1.44/ 1.43/1.43	**0.27/ 0.31/0.35**	**–0.19/ -0.20/-0.20**
Mulliken (DZ/ TZ/QZ)	0.89/ 0.79/1.23	1.32/ 1.23/1.46	1.15/ 0.92/1.31	**0.23/ 0.13/0.09**	**–0.19/ -0.30/-0.15**

Copper (I)

A) Linear symmetry of GCN–Cu^+

Table S18 Bond distances M–L (L = O, N) of optimized structures (in Å) of $[Cu(H_2O)_2]^+$, GCN–$[Cu(H_2O)]^+$, and ACN–$[Cu(H_2O)]^+$ complexes.

**Bond**	**System**		
	$[Cu(H_2O)_2]^+$	GCN–$[Cu(H_2O)]^+$	ACN–$[Cu(H_2O)]^+$
Cu–O_1	1.940	1.955	1.949
Cu–O_2	1.940	–	–
Cu–N	–	**1.854**	**1.853**
C–CN	–	1.157	1.157

Table S19 Occupancies of valence orbitals of respective Cu(I) complexes (linear arrangement), including the total occupancy as well as the natural and Mulliken charges of the central ion obtained at the PBE0/def2-SVPP level. For comparison, the total charges obtained using the def2-TZVP and def2-QZVP basis sets are reported.

Orbitals Cu^+	System			Difference	
	$[Cu(H_2O)_2]^+$	Fragment –$[Cu(H_2O)]^+$	GCN–$[Cu(H_2O)]^+$	GCN–$[Cu(H_2O)]^+$ vs $[Cu(H_2O)_2]^+$	GCN–$[Cu(H_2O)]^+$ vs frag–$[Cu(H_2O)]^+$
$3d_{z2}$	1.947	1.889	1.841	–0.106	–0.048
$3d_{x2-y2}$	1.843	1.999	1.999	0.156	0.000
$3d_{yz}$	1.999	1.997	1.954	–0.045	–0.043
$3d_{xz}$	1.997	1.998	1.952	–0.045	–0.046
$3d_{xy}$	1.996	1.999	1.999	0.003	0.000
4s	0.380	0.163	0.352	–0.028	0.189
$4p_x$	0.026	0.002	0.006	–0.020	0.004
$4p_y$	0.002	0.000	0.003	0.001	0.003
$4p_z$	0.007	0.005	0.038	0.031	0.033
Total	10.20	10.05	10.14	**–0.05**	**0.09**
Natural (DZ/ TZ/QZ)	0.80/ 0.82/0.83	0.94/ 0.96/0.96	0.85/ 0.86/0.86	**0.06/ 0.04/0.03**	**–0.09/ -0.10/-0.10**
Mulliken (DZ/ TZ/QZ)	0.60/ 0.56/0.72	0.81/ 0.85/0.88	0.65/ 0.47/0.71	**0.05/ -0.09/0.00**	**–0.16/ -0.38/-0.16**

(B) Trigonal planar symmetry of GCN–Cu^+

Table S20 Bond distances M–L (L = O, N) of optimized structures (in Å) of GCN–$[Cu(H_2O)_2]^+$, and ACN–$[Cu(H_2O)_2]^+$ complexes.

**Bond**	**System**	
	GCN–$[Cu(H_2O)_2]^+$	ACN–$[Cu(H_2O)_2]^+$
Cu–O_1	2.082	2.074
Cu–O_2	2.104	2.118
Cu–N	**1.865**	**1.865**
C–CN	1.160	1.159

Table S21 Occupancies of valence orbitals of respective Cu(I) complexes (Trigonal Planar symmetry), including the total occupancy as well as the natural and Mulliken charges of the central ion obtained at the PBE0/def2-SVPP level. For comparison, the total charges obtained using the def2-TZVP and def2-QZVP basis sets are reported.

Orbitals Cu^+	System			Difference	
	$[Cu(H_2O)_2]^+$	Fragment –$[Cu(H_2O)_2]^+$	GCN– $[Cu(H_2O)_2]^+$	GCN– $[Cu(H_2O)_2]^+$ vs $[Cu(H_2O)_2]^+$	GCN– $[Cu(H_2O)_2]^+$ vs frag–$[Cu(H_2O)_2]^+$
$3d_{z2}$	1.947	1.985	1.918	–0.029	–0.067
$3d_{x2-y2}$	1.843	1.998	1.999	0.156	0.001
$3d_{yz}$	1.999	1.993	1.931	–0.068	–0.062
$3d_{xz}$	1.997	1.995	1.935	–0.062	–0.060
$3d_{xy}$	1.996	1.963	1.988	–0.008	0.025
4s	0.380	0.119	0.272	–0.108	0.153
$4p_x$	0.026	0.014	0.031	0.005	0.017
$4p_y$	0.002	0.017	0.036	0.034	0.019
$4p_z$	0.007	0.007	0.076	0.069	0.069
Total	10.20	10.09	10.19	**–0.01**	**0.10**
Natural (DZ/ TZ/QZ)	0.80/ 0.82/0.83	0.91/ 0.93/0.93	0.81/ 0.81/0.81	**0.01/ -0.01/-0.02**	**–0.10/ -0.12/-0.12**
Mulliken (DZ/ TZ/QZ)	0.60/ 0.56/0.72	0.71/ 0.76/0.81	0.57/ 0.53/0.70	**–0.03/ -0.03/-0.01**	**–0.14/ -0.23/-0.10**

Copper (II)

Table S22 Bond distances M–L (L = O, N) of optimized structures (in Å) of $[Cu(H_2O)_6]^{2+}$, GCN–$[Cu(H_2O)_3]^{2+}$, and ACN–$[Cu(H_2O)_5]^{2+}$ complexes.

**Bond**	**System**			
	$[Cu(H_2O)_6]^{2+}$ (Oh symmetry)	GCN–$[Cu(H_2O)_3]^{2+}$ (Th symmetry)	GCN–$[Cu(H_2O)_3]^{2+}$ (Tg symmetry)	ACN–$[Cu(H_2O)_3]^{2+}$ (Tg symmetry)
Cu–O_1	2.054	2.144	1.995	1.993
Cu–O_2	2.048	2.296	1.988	2.005
Cu–O_3	2.058	2.222	2.023	2.015
Cu–O_4	2.052	–	–	–
Cu–O_5	2.357	–	–	–
Cu–O_6	2.332	–	–	–
Cu–N	–	**1.864**	**2.044**	**2.059**
C–CN	–	1.159	1.153	1.153

(A) Tetrahedral symmetry of GCN–Cu^{2+}

Table S23 Occupancies of valence orbitals of respective Cu(II) complexes (Th), including the total occupancy as well as the natural and Mulliken charges of the central ion obtained at the PBE0/def2-SVPP level. For comparison, the total charges obtained using the def2-TZVP and def2-QZVP basis sets are reported.

Orbitals Cu^{2+}	System			Difference	
	$[Cu(H_2O)_6]^{2+}$	Fragment –$[Cu(H_2O)_3]^{2+}$	GCN–$[Cu(H_2O)_3]^{2+}$	GCN–$[Cu(H_2O)_3]^{2+}$ vs $[Cu(H_2O)_6]^{2+}$	GCN–$[Cu(H_2O)_3]^{2+}$ vs frag–$[Cu(H_2O)_3]^{2+}$
$3d_{z2}$	1.995	1.983	1.931	–0.064	–0.052
$3d_{x2-y2}$	1.180	1.878	1.997	0.817	0.119
$3d_{yz}$	1.998	1.350	1.924	–0.074	0.574
$3d_{xz}$	1.995	1.927	1.924	–0.071	–0.003
$3d_{xy}$	1.996	1.949	1.996	0	0.047
4s	0.267	0.105	0.259	–0.008	0.154
$4p_x$	0.111	0.019	0.043	–0.068	0.024
$4p_y$	0.108	0.020	0.052	–0.056	0.032
$4p_z$	0.090	0.014	0.092	0.002	0.078
Total	9.74	9.25	10.22	**0.48**	**0.97**
Natural (DZ/ TZ/QZ)	1.25/ 1.26/1.25	1.75/ 1.79/1.79	0.78/ 0.78/0.76	**–0.48/ -0.49/-0.49**	**–0.97/ -1.01/-1.03**
Mulliken (DZ/ TZ/QZ)	0.89/ 0.81/1.36	1.45/ 1.49/1.66	0.52/ 0.45/0.56	**–0.37/ -0.36/-0.79**	**–0.93/ -1.04/-1.10**

(B) Tetragonal symmetry of GCN–Cu^{2+}

Table S24 Occupancies of valence orbitals of respective Cu(II) complexes (Tg), including the total occupancy as well as the natural and Mulliken charges of the central ion obtained at the PBE0/def2-SVPP level. For comparison, the total charges obtained using the def2-TZVP and def2-QZVP basis sets are reported.

Orbitals Cu^{2+}	System			Difference	
	$[Cu(H_2O)_6]^{2+}$	Fragment –$[Cu(H_2O)_3]^{2+}$	GCN–$[Cu(H_2O)_3]^{2+}$	GCN–$[Cu(H_2O)_3]^{2+}$ vs $[Cu(H_2O)_6]^{2+}$	GCN–$[Cu(H_2O)_3]^{2+}$ vs frag–$[Cu(H_2O)_3]^{2+}$
$3d_{z2}$	1.995	1.502	1.442	–0.553	–0.060
$3d_{x2-y2}$	1.180	1.650	1.752	0.572	0.102
$3d_{yz}$	1.998	1.997	1.992	–0.006	–0.005
$3d_{xz}$	1.995	1.998	1.994	–0.001	–0.004
$3d_{xy}$	1.996	1.994	1.996	0	0.002
4s	0.267	0.158	0.235	–0.032	0.077
$4p_x$	0.111	0.055	0.090	–0.021	0.035
$4p_y$	0.108	0.011	0.019	–0.089	0.008
$4p_z$	0.090	0.029	0.095	0.005	0.066
Total	9.74	9.35	9.62	**–0.13**	**0.22**
Natural (DZ/ TZ/QZ)	1.25/ 1.26/1.25	1.60/ 1.63/1.64	1.38/ 1.39/1.41	**0.13/ 0.13/0.16**	**–0.22/ -0.23/-0.23**
Mulliken (DZ/ TZ/QZ)	0.89/ 0.81/1.36	1.30/ 1.20/1.48	1.07/ 0.74/1.23	**0.18/ -0.07/-0.12**	**–0.24/ -0.46/-0.25**

Zinc (Zn^{2+})

Table S25 Bond distances M–L (L = O, N) of optimized structures (in Å) of $[Zn(H_2O)_6]^{2+}$, GCN–$[Zn(H_2O)_6]^{2+}$, and ACN–$[Zn(H_2O)_5]^{2+}$ complexes.

Bond	System		
	$[Zn(H_2O)_6]^{2+}$	GCN–$[Zn(H_2O)_5]^{2+}$	ACN–$[Zn(H_2O)_5]^{2+}$
Zn–O_1	2.174	2.124	2.142
Zn–O_2	2.178	2.155	2.167
Zn–O_3	2.172	2.102	2.175
Zn–O_4	2.158	2.135	2.137
Zn–O_5	2.164	2.161	2.216
Zn–O_6	2.171	–	–
Zn–N	–	**2.601**	**2.218**
C–CN	–	1.157	1.155

Table S26 Occupancies of valence orbitals of respective Zn(II) complexes, including the total occupancy as well as the natural and Mulliken charges of the central ion obtained at the PBE0/def2-SVPP level. For comparison, the total charges obtained using the def2-TZVP and def2-QZVP basis sets are reported.

Orbitals Zn^{2+}	System			Difference	
	$[Zn(H_2O)_6]^{2+}$	Fragment –$[Zn(H_2O)_5]^{2+}$	GCN–$[Zn(H_2O)_5]^{2+}$	GCN–$[Zn(H_2O)_5]^{2+}$ vs $[Zn(H_2O)_6]^{2+}$	GCN–$[Zn(H_2O)_5]^{2+}$ vs frag–$[Zn(H_2O)_5]^{2+}$
$3d_{z2}$	1.998	1.995	1.995	–0.003	0.000
$3d_{x2-y2}$	1.998	1.988	1.988	–0.010	0.000
$3d_{yz}$	1.998	1.988	1.988	–0.010	0.000
$3d_{xz}$	1.997	1.988	1.988	–0.009	0.000
$3d_{xy}$	1.998	1.988	1.988	–0.010	0.000
4s	0.264	0.240	0.262	–0.002	0.022
$4p_x$	0.097	0.083	0.098	–0.001	0.015
$4p_y$	0.096	0.085	0.096	0.000	0.011
$4p_z$	0.096	0.060	0.083	–0.013	0.023
Total	10.54	10.42	10.49	**-0.06**	**0.07**
Natural (DZ/ TZ/QZ)	1.45/ 1.49/1.37	1.54/ 1.58/1.51	1.47/ 1.49/1.38	**0.02/ 0.01/0.02**	**-0.07/ -0.08/-0.13**
Mulliken (DZ/ TZ/QZ)	1.10/ 1.07/1.51	1.21/ 1.16/1.54	1.11/ 1.07/1.53	**0.01/ 0.00/0.02**	**–0.10/ -0.10/-0.01**

Table S27 Impact of a ligand (GCN or ACN) on the amount of transferred charge in the GCN/ACN−Me complexes. The population analysis based on natural charges was performed at the PBE0/def2-QZVP level.

Me^{x+}	Final symmetry of complex	Mulliken charges on Me^{x+}					
		**Fragment**	**GCN**	**Δ*q***	**Fragment**	**ACN**	**Δ*q***
Fe^{2+}	Oh	1.49	1.26	–0.23	1.52	1.26	–0.26
Fe^{3+}	Oh	2.17	1.32	–0.85	–	–	–
Co^{2+}	Oh	1.45	1.18	–0.27	1.45	1.18	–0.27
Co^{2+}	Th	1.73	1.49	–0.23	1.73	1.49	–0.23
Ni^{2+}	Oh	1.31	1.03	–0.28	1.31	1.02	–0.29
Ni^{2+}	Tg	1.64	1.43	–0.20	–	–	–
Cu^{+}	Lin	0.96	0.86	–0.10	0.96	0.84	–0.13
Cu^{+}	Trig.pl	0.93	0.81	–0.12	0.93	0.79	–0.14
Cu^{2+}	Th	1.79	0.76	–1.03	–	–	–
Cu^{2+}	Tg	1.64	1.41	–0.23	1.64	1.42	–0.23
Zn^{2+}	Oh	1.51	1.38	–0.13	1.53	1.31	–0.22

Additional experimental data

Determination of functionalization degree of GCN:

In order to experimentally determine thermodynamic characteristics of the Cu^{2+} coordination on GCN, functionalization degree of GCN needed to be determined to know what quantity of CN groups is in certain mass of GCN. This was achieved through the same rational used in the work of Bakandritsos *et al.* in the reference.[2] For this, we analyzed the graphene acid (GA) sample synthesized from the same batch of GCN used in this study.

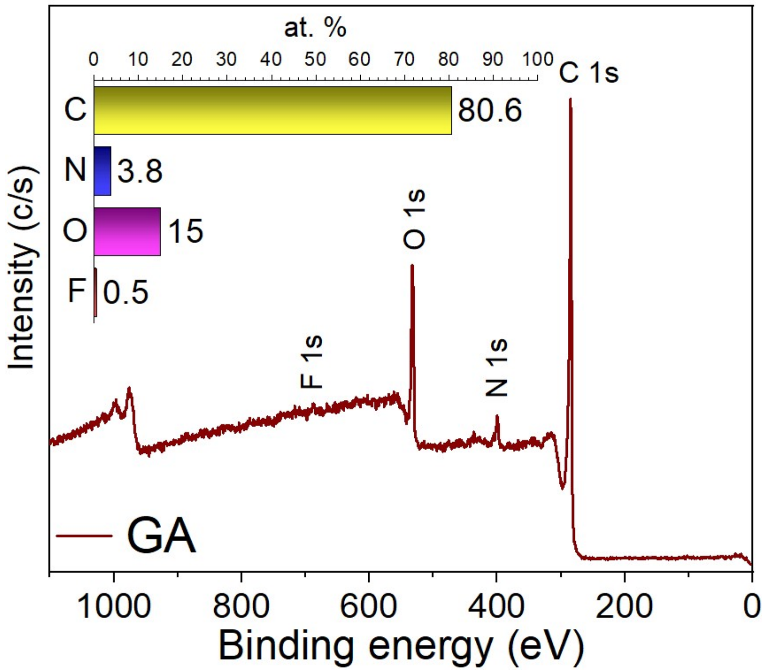

Figure S1: Survey XPS analyses of GCN and graphene acid (GA) synthesized thereof with determined atomic compositions.

According to XPS analysis (Figure S3a) in GCN there is 12.9 at. % of N. Residual 3.8 at. % of N found in GA (Figure S1) is considered as contaminant not related to CN groups. Therefore. there is 9.1 at. % of CN groups in our GCN material. The formula representing the composition of our GCN therefore is:

$$(CN)_{9.1}N_{3.8}C_{71.9}O_{4.3}F_{1.4}$$

The molecular weight of this hypothetical unit therefore is:

$$M_W = 1248\ g{\cdot}mol^{-1}.$$

from which 236.6 $g{\cdot}mol^{-1}$ corresponds to CN groups alone. Therefore, the mass fraction of CN groups in our GCN is:

$$w_{CN} = \frac{M_W(CN)}{M_W} = \frac{236.6}{1248} = 0.1896$$

which practically is **19 wt. % of CN groups in our GCN**.

1 mg of GCN therefore contains **0.19 mg (7.31 µmol) of CN** groups, dispersion containing **1 mg/mL of GCN** was therefore in ITC experiments regarded as **7.31mM CN solution**.

Monitoring of GCN-Me samples washing

The monitoring of was done to observe the trend how the residual unbonded metal ions were released from the material during their washings. The volume of water used for each washing step was the same as used for the immobilization of the metal. The time of redispersion of the filtered material was not fixed, but usually within several minutes. Considering the final adsorbed amounts of metals on the GCN, the amounts of metals in all the analyzed filtrates were overall in all cases lower than the initial amounts due to due to adventitious dilution of the filtrates and possible metal sorption on the filtration apparatus.

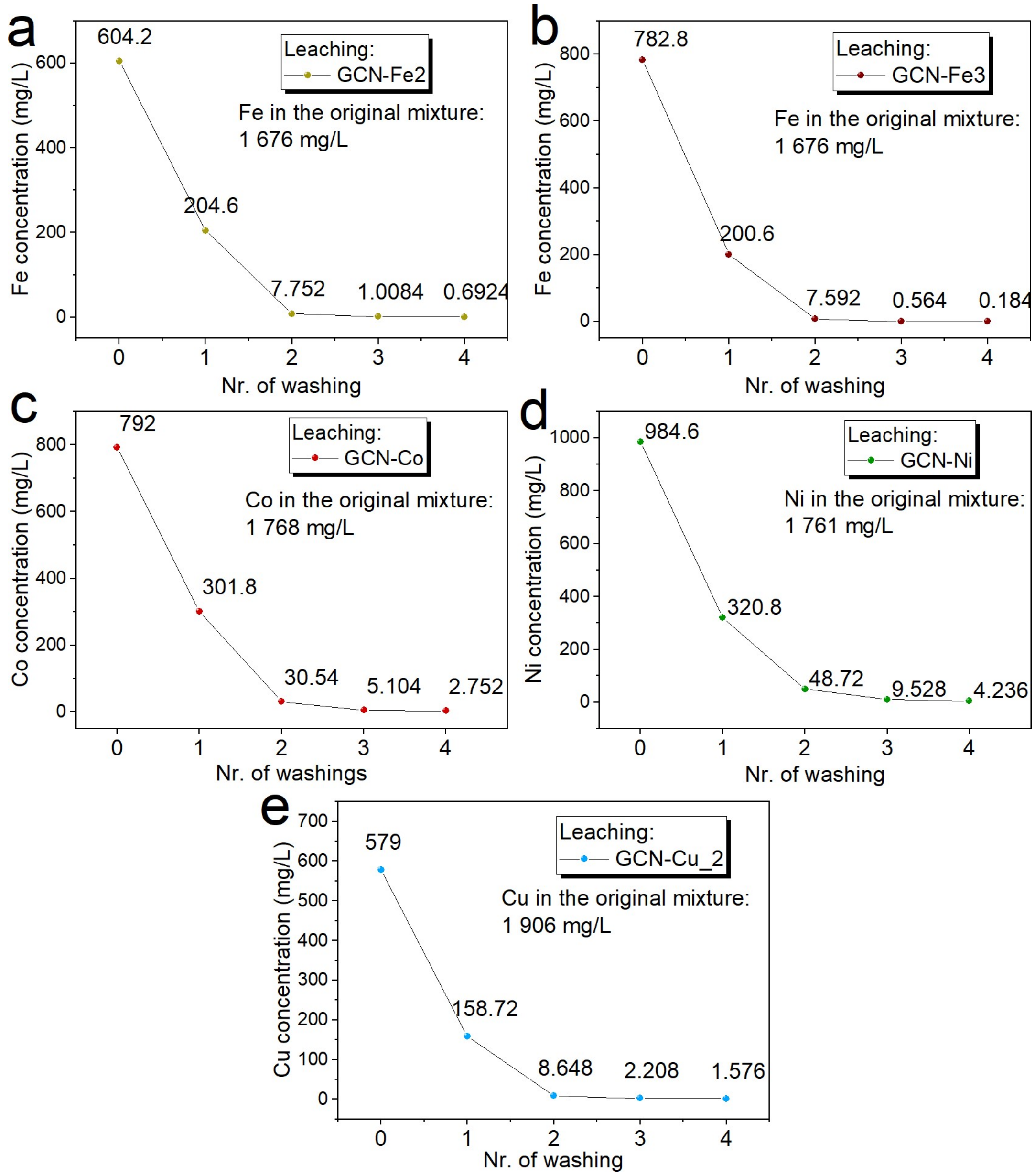

Figure S2: Leaching of metals form the GCN-M hybrids during each washing step as metal concentrations in the filtrates in the case of GCN with (a) Fe(II), (b) Fe(III), (c) Co(II), (d) Ni(II), (e) Cu(II) (2nd batch), as determined using AAS measurements.

XPS survey data and determined atomic compositions of the analysed materials

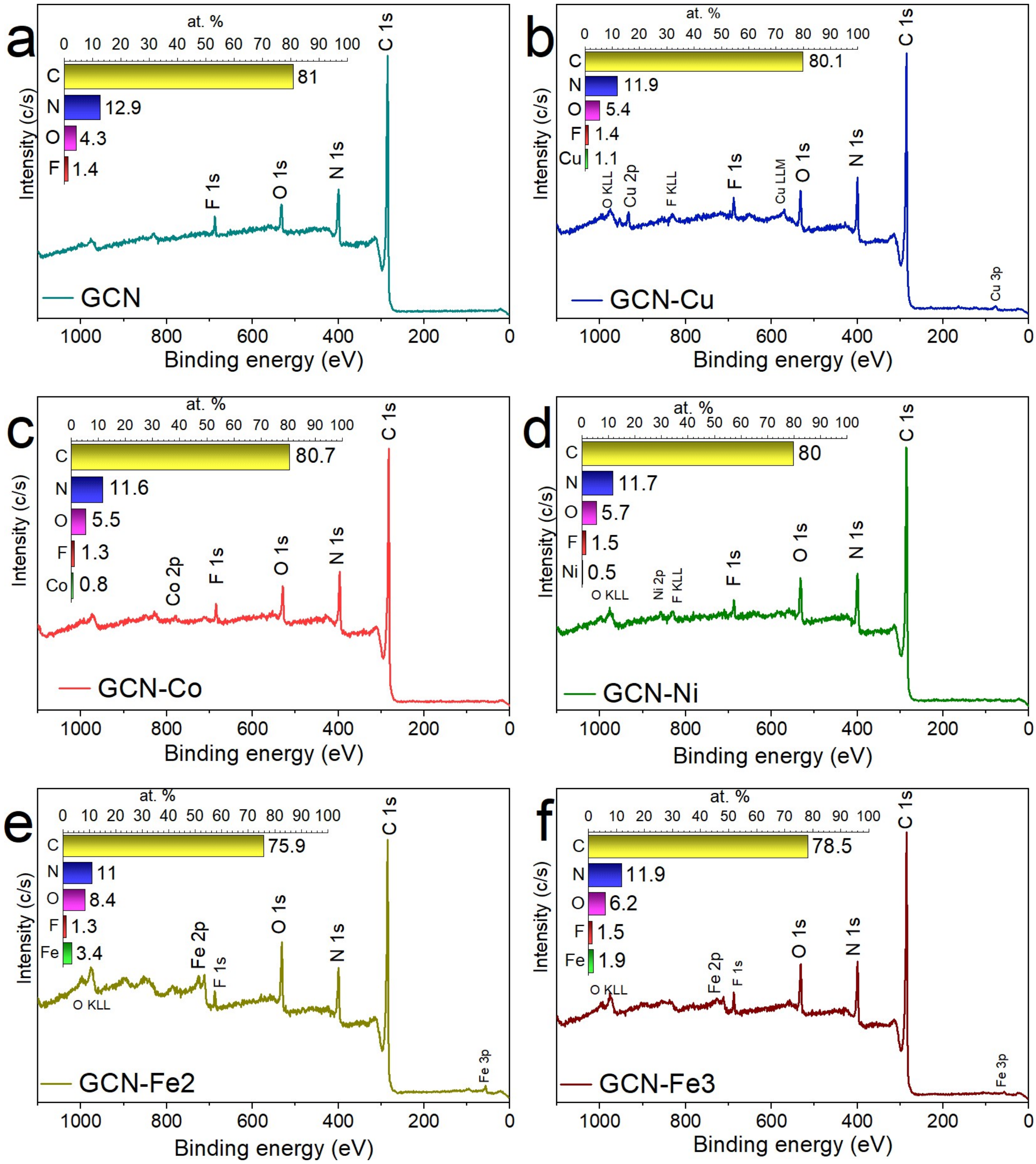

Figure S3: Survey XPS spectra of pristine GCN (a), GCN with Cu (first batch) (b), Co (c), Ni (d), Fe(II) (e), and Fe(III) (f). Determined atomic compositions of each material are shown in the inset of each panel.

X-ray diffraction of GCN-Fe samples

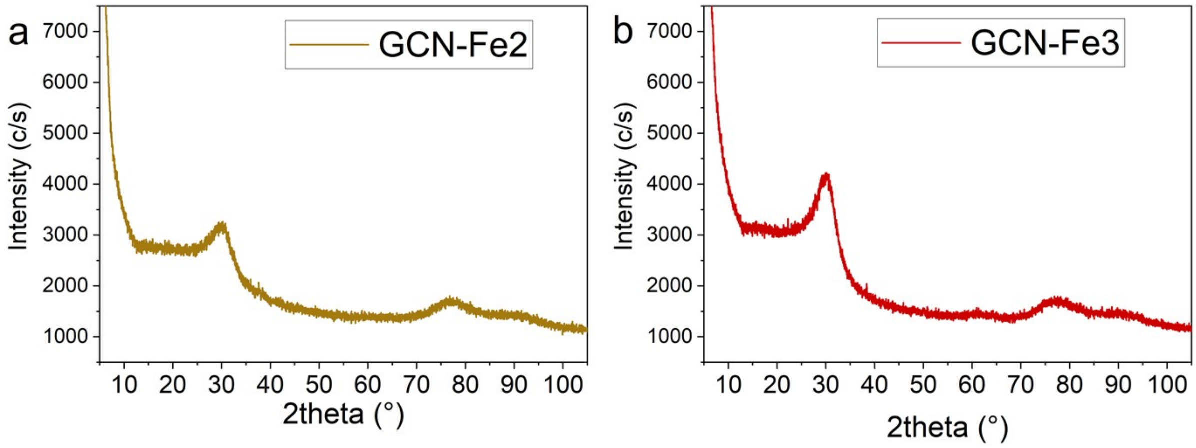

Figure S4: XRD patterns of GCN-Fe2 (a) and GCN-Fe3 (b) samples recorded on a PANalytical X'Pert PRO diffractometer (iron-filtered Co Kα radiation: λ=0.178901 nm, 40 kV, and 30 mA) in the Bragg–Brentano geometry, equipped with an X'Celerator detector, programmable divergence and diffracted beam anti-scatter slits. A uniform layer of each sample was created on a zero-background Si slide by drop-casting the GCN-Fe material dispersion and scanned with a step size of 0.0334°, and 2θ scan range from 5° to 105°.

Isothermal titration calorimetry

The ITC experiments were performed at the temperature of 25 °C. Overall, three measurements were carried out, in which 800 µL of GCN dispersion (twice with the concentration of 3.125 mg/mL which is equivalent to 22.84 mM in terms of CN groups, once as 7.6mM CN solution) was titrated with $Cu(NO_3)_2$ solution by injecting 60 x 3 µL of 50mM solution, 60 x 3 µL of 37.5mM solution, and 50 x 4 µL of 10mM solution, respectively. The time interval between each injection was 5 minutes.

In all three experiments (Figure S5 a, c, e), initial injections of Cu^{2+} into GCN resulted in a sudden exothermic response that could be attributed to the interactions of Cu ions with highly reactive sites such as radical defects and vacancies in the GCN structure. With more injections, the response changed to endothermic, which after peaking gradually flipped to a slightly exothermic regime upon continued injection of Cu^{2+}. Since the endothermic response occurred in a shorter timescale when higher concentration of Cu^{2+} solution was used for the titration (cf. panels a and c in Figure S6), the endothermic response was linked to the immobilization of copper ions on the CN groups of GCN. After the saturation of the CN groups, the slightly exothermic processes after each injection that occurred consistently till the end of the measurement was attributed to Cu^{2+} ions diluting in the mixture.

The obtained data were fitted using the *independent model* (panels b, d, and f in Figure S5) as implemented in NanoAnalyze Data Analysis software ver. 3.12.00. Although the *multiple sites model* fitted the initial part of the enthalpic data better, it provided enthalpy values with extremely high standard deviations indicating the numerical instability of the model, unlike the independent model. The discrepancy between the independent fit and the initial part of the data was caused by the fact that the exothermic process took place in the initial phase of injecting. To check the sensitivity of the independent model, we performed a series of fits excluding up to four initial recorded points. The derived thermodynamic quantities for experiments 1-3 as a function of the number of excluded points are listed in Tables S28-S30, respectively. The mean values of the thermodynamic quantities with their standard deviations are listed in Table S31 for each experiment along with values determined from all the determined values in the last row of the table.

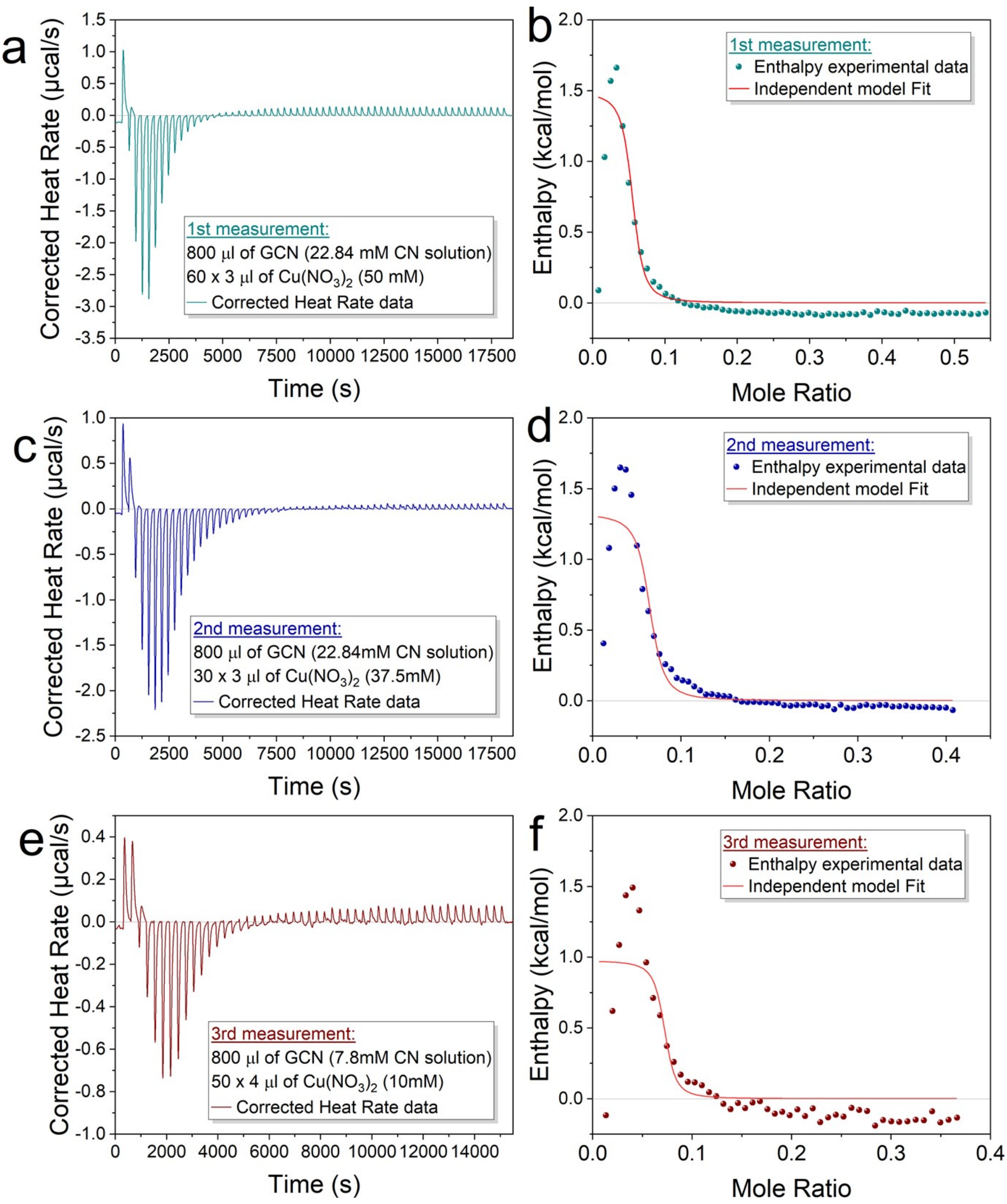

Figure S5: Isothermal titration calorimetry results from three performed experiments. Corrected heated rate data measured in the first (a), second (c), and third experiment (e), and plotted enthalpy/molar ratio dependencies generated from the heat rate data of the first (b), second (d), and third experiment (f) and fitted using the independent model, which afforded the thermodynamic quantities that can be found in Tables S28-S31.

Table S28: Thermodynamic characteristics of Cu^{2+} immobilization on GCN according to independent fit of ITC data from the first measurement for cases, when the first and first two initial points from the data were excluded.

1st measurement	*ΔH*	*-TΔS*	*ΔG*	*ΔS*
Nr. of excluded initial points	*(kcal/mol)*	*(kcal/mol)*	*(kcal/mol)*	*(cal/mol · K)*
1	1.5	-7.7	-6.2	25.9
2	1.8	-7.8	-5.9	26.1

Table S29: Thermodynamic characteristics of Cu^{2+} immobilization on GCN according to independent fit of ITC data from the second measurement for cases, when the first, first two, and first three initial points from the data were excluded.

2nd measurement	*ΔH*	*-TΔS*	*ΔG*	*ΔS*
Nr. of excluded initial points	*(kcal/mol)*	*(kcal/mol)*	*(kcal/mol)*	*(cal/mol · K)*
1	1.3	-7.6	-6.3	25.6
2	1.6	-7.6	-6.0	25.6
3	1.8	-7.7	-5.8	25.8

Table S30: Thermodynamic characteristics of Cu^{2+} immobilization on GCN according to independent fit of ITC data from the first measurement for cases, when the first, first two, first three, and first four initial points from the data were excluded.

3rd measurement	*ΔH*	*-TΔS*	*ΔG*	*ΔS*
Nr. of excluded initial points	*(kcal/mol)*	*(kcal/mol)*	*(kcal/mol)*	*(cal/mol · K)*
1	0.98	-8.4	-7.5	28.3
2	1.2	-8.3	-7.1	28.0
3	1.4	-8.3	-6.9	27.9
4	1.7	-8.3	-6.7	28.0

Table S31: Mean values of thermodynamic characteristics of Cu^{2+} immobilization on GCN according to independent fits of ITC data from individual measurements including the mean values acquired from all the measurements and fits.

Nr. of experiment	*ΔH* *(kcal/mol)*	*-TΔS* *(kcal/mol)*	*ΔG* *(kcal/mol)*	*ΔS* *(cal/mol · K)*
1	1.7 ± 0.3	-7.8 ± 0.1	-6.1 ± 0.2	26.0 ± 0.2
2	1.6 ± 0.3	-7.7 ± 0.1	-6.1 ± 0.2	25.7 ± 0.1
3	1.3 ± 0.3	-8.4 ± 0.1	-7.0 ± 0.3	28.0 ± 0.2
all	1.5 ± 0.3	-8.0 ± 0.4	-6.5 ± 0.6	26.8 ± 1.2

References

(1) Zaoralová, D.; Mach, R.; Lazar, P.; Medveď, M.; Otyepka, M. Anchoring of Transition Metals to Graphene Derivatives as an Efficient Approach for Designing Single-Atom Catalysts. *Adv. Mater. Interfaces* **2021**, *8* (8), 2001392. https://doi.org/10.1002/admi.202001392.
(2) Bakandritsos, A.; Pykal, M.; Błoński, P.; Jakubec, P.; Chronopoulos, D. D.; Poláková, K.; Georgakilas, V.; Čépe, K.; Tomanec, O.; Ranc, V.; Bourlinos, A. B.; Zbořil, R.; Otyepka, M. Cyanographene and Graphene Acid: Emerging Derivatives Enabling High-Yield and Selective Functionalization of Graphene. *ACS Nano* **2017**, *11* (3), 2982–2991. https://doi.org/10.1021/acsnano.6b08449.
